# *Candidatus* Nitrosopolaris, a genus of putative ammonia-oxidizing archaea with a polar/alpine distribution

**DOI:** 10.1093/femsmc/xtac019

**Published:** 2022-06-24

**Authors:** Igor S Pessi, Aino Rutanen, Jenni Hultman

**Affiliations:** Department of Microbiology, University of Helsinki, Viikinkaari 9, 00014 Helsinki, Finland; Helsinki Institute of Sustainability Science (HELSUS),Yliopistonkatu 3, 00014 Helsinki, Finland; Department of Microbiology, University of Helsinki, Viikinkaari 9, 00014 Helsinki, Finland; Department of Microbiology, University of Helsinki, Viikinkaari 9, 00014 Helsinki, Finland; Helsinki Institute of Sustainability Science (HELSUS),Yliopistonkatu 3, 00014 Helsinki, Finland; Natural Resources Institute Finland (LUKE),Latokartanonkaari 9, 00790 Helsinki, Finland

**Keywords:** ammonia oxidation, archaea, climate change, metagenome assembled genomes, polar microbes, tundra

## Abstract

Ammonia-oxidizing archaea (AOA) are key players in the nitrogen cycle of polar soils. Here, we analyzed metagenomic data from tundra soils in Rásttigáisá, Norway, and recovered four metagenome-assembled genomes (MAGs) assigned to the genus ‘UBA10452’, an uncultured lineage of putative AOA in the order Nitrososphaerales (‘terrestrial group I.1b’), phylum Thaumarchaeota. Analysis of other eight previously reported MAGs and publicly available amplicon sequencing data revealed that the UBA10452 lineage is predominantly found in acidic polar and alpine soils. In particular, UBA10452 MAGs were more abundant in highly oligotrophic environments such as mineral permafrost than in more nutrient-rich, vegetated tundra soils. UBA10452 MAGs harbour multiple copies of genes related to cold tolerance, particularly genes involved in DNA replication and repair. Based on the phylogenetic, biogeographic, and ecological characteristics of 12 UBA10452 MAGs, which include a high-quality MAG (90.8% complete, 3.9% redundant) with a nearly complete 16S rRNA gene, we propose a novel *Candidatus* genus, *Ca*. Nitrosopolaris, with four species representing clear biogeographic/habitat clusters.

## Teaser

Tundra soils harbour novel ammonia-oxidizing archaea

## Introduction

Nitrification—the oxidation of ammonia to nitrite and further oxidation to nitrate—is a crucial part of the nitrogen (N) cycle providing a link between reduced and oxidized forms of N. The first step of nitrification, ammonia oxidation, is carried out mainly by aerobic chemolithoautotrophic microorganisms that grow by coupling the energy obtained from the oxidation of ammonia with carbon dioxide (CO_2_) fixation (Lehtovirta-Morley 2018). Ammonia-oxidizing archaea (AOA) outnumber ammonia-oxidizing bacteria (AOB) by orders of magnitude in many terrestrial and aquatic environments, particularly in oligotrophic environments with low N input (Leininger et al. 2006, Schleper and Nicol 2010, Lehtovirta-Morley 2018). Among the reasons for their ecological success is an enzymatic machinery with higher affinity for ammonia and a more efficient CO_2_ fixation pathway than their bacterial counterparts (Martens-Habbena et al. 2009, Könneke et al. 2014, Kerou et al. 2016). However, high ammonia affinity is not a common trait to all AOA, with some strains displaying a low substrate affinity that is comparable to that of nonoligotrophic AOB (Kits et al. 2017, Jung et al. 2022).

Ammonia oxidation is an important process in polar soils despite commonly N limited and cold conditions (Alves et al. 2013, Siljanen et al. 2019, Hayashi et al. 2020). AOA generally outnumber AOB in oligotrophic polar soils and are often represented by few species (Alves et al. 2013, Magalhães et al. 2014, Richter et al. 2014, Pessi et al. 2015, 2022, Siljanen et al. 2019, Ortiz et al. 2020). Due to their predominance, AOA are important contributors to the N cycle in polar soils and thus are key players in the cycling of the potent greenhouse gas nitrous oxide (N_2_O). Contrary to earlier assumptions, polar soils are increasingly recognized as important sources of N_2_O (Voigt et al. 2020). Both the nitrite originated from the oxidation of ammonia as well as the nitrate produced in the second step of nitrification are the substrates of denitrification, an anaerobic process that has N_2_O as a gaseous intermediate (Butterbach-Bahl et al. 2013). Moreover, AOA have been directly implicated in the production of N_2_O under oxic conditions via several mechanisms such as hydroxylalamine oxidation and nitrifier denitrification (Wu et al. 2020). However, both the direct and indirect roles of AOA in the cycling of N_2_O are much less understood compared to their bacterial counterparts.

AOA are notoriously difficult to cultivate, and so far only three genera have been formally described based on axenic cultures: *Nitrosopumilus* (Qin et al. 2017) and *Nitrosarchaeum* (Jung et al. 2018) in the order Nitrosopumilales (‘marine group I.1a’) and *Nitrososphaera* (Stieglmeier et al. 2014) in the order Nitrososphaerales (‘terrestrial group I.1b’). Several provisional *Candidatus* genera have also been proposed based on nonaxenic enrichments, e.g. *Ca*. Nitrosocaldus (‘thermophilic group’; de la Torre et al. 2008) and *Ca*. Nitrosotalea (‘group I.1a-associated’; Lehtovirta-Morley et al. 2011). Moreover, the growing use of genome-resolved metagenomics has resulted in the identification of tens of novel, currently uncultured lineages in the phylum Thaumarchaeota. These lineages are phylogenetically distinct from both formally described and *Candidatus* taxa and are normally labelled with placeholder alphanumeric identifiers (e.g. the Nitrososphaerales genus ‘UBA10452’; Rinke et al. 2021). The discovery of these novel lineages by metagenomics greatly expands our knowledge of the diversity of AOA, but detailed descriptions of their metabolic and ecological features are generally lacking.

Recently, we have applied a genome-resolved metagenomics approach to gain insights into the microorganisms involved with the cycling of greenhouse gases in tundra soils in Kilpisjärvi, Finland (Pessi et al. 2022). Analysis of *amoA* genes encoding the alpha subunit of the enzyme ammonia monooxygenase (Amo) revealed a very low diversity of ammonia oxidizers, with only four genes annotated as *amoA* out of 23.5 million assembled genes. Three of these were most closely related to the *amoA* gene of the comammox bacterium *Ca*. Nitrospira inopinata (Daims et al. 2015). The remaining *amoA* gene was binned into a metagenome-assembled genome (MAG) assigned to the genus ‘UBA10452’, an uncharacterized archaeal lineage in the order Nitrososphaerales (Rinke et al. 2021). Here, we (i) report four novel UBA10452 MAGs obtained from tundra soil in Rásttigáisá, Norway; (ii) characterize the genomic properties, metabolic potential, phylogeny, and biogeography of the UBA10452 lineage; and (iii) propose the creation of a new *Candidatus* genus, *Ca*. Nitrosopolaris.

## Methods

### Sampling and metagenome sequencing

A total of 10 soil samples were obtained in July 2017 across an area of alpine tundra in Rásttigáisá, Norway (69 59 N, 26 15 E, and 700 m.a.s.l.). DNA was extracted from the mineral layer (10–15 cm depth) with the PowerSoil DNA Isolation kit (QIAGEN, Venlo, Netherlands) according to the manufacturer's instructions. Paired-end metagenomic sequencing was done using the Illumina NextSeq500 platform (Illumina, San Diego, CA, USA) at the DNA Sequencing and Genomics Laboratory (Institute of Biotechnology, University of Helsinki).

### Metagenome assembling and binning

Removal of adapter sequences and low-quality base calls (Phred score < 28) was done with Cutadapt v1.10 (Martin 2011) and sequences were assembled with MEGAHIT v1.1.1 setting a minimum contig length of 1000 bp (Li et al. 2015). Samples were assembled individually and as one coassembly of all samples pooled together. Manual MAG binning was done with anvi'o v6.0 (Eren et al. 2015) according to Pessi et al. (2022). In brief, Prodigal v2.6.3 (Hyatt et al. 2010) was used to predict gene calls and single-copy genes were identified with HMMER v.3.2.1 (Eddy 2011). Bowtie v2.3.5 (Langmead and Salzberg 2012) and SAMtools v1.9 (Li et al. 2009) were used to map the quality-filtered Illumina reads to the contigs. Contigs were then manually binned into MAGs based on differential coverage and tetranucleotide frequency using the *anvi-interactive* interface of anvi'o v6.0. MAGs were manually inspected and refined using the *anvi-refine* interface of anvi'o v6.0.

### MAGs assigned to the UBA14052 lineage

MAGs were classified based on 122 archaeal and 120 bacterial single-copy genes with GTDB-Tk v1.3.0 (Chaumeil et al. 2020) and the GTDB release 05-RS95 (Parks et al. 2018, 2020). MAGs assigned to the genus ‘UBA10452’ in the order Nitrososphaerales (Rinke et al. 2021), were selected for downstream analyses (Table 1; Table S1, Supporting Information). In addition, we analyzed other eight UBA10452 MAGs available on GenBank. These included six MAGs from permafrost soil in Canada (Chauhan et al. 2014, Parks et al. 2017), one MAG from polar desert soil in Antarctica (Ji et al. 2017), and one MAG from tundra soil in Finland (Pessi et al. 2022).

**Table 1.** List of MAGs belonging to the UBA10452 lineage (*Ca*. Nitrosopolaris).

MAG	Isolation source	Accession	Ref.
COA_Bin_4_1	Tundra soil, Rásttigáisá, Norway	GCA_933227015.1	[1]
S89_Bin_2	Tundra soil, Rásttigáisá, Norway	GCA_933227005.1	[1]
S100_Bin_4	Tundra soil, Rásttigáisá, Norway	GCA_933226995.1	[1]
S1130_Bin_3	Tundra soil, Rásttigáisá, Norway	GCA_933226985.1	[1]
KWL-0179	Tundra soil, Kilpisjärvi, Finland	GCA_936417005.1	[2]
UBA272	Permafrost soil, Nunavut, Canada	GCA_002504425.1	[3]
UBA273	Permafrost soil, Nunavut, Canada	GCA_002501935.1	[3]
UBA347	Permafrost soil (active layer), Nunavut, Canada	GCA_002495965.1	[3]
UBA348	Permafrost soil (active layer), Nunavut, Canada	GCA_002501855.1	[3]
UBA466	Permafrost soil (active layer), Nunavut, Canada	GCA_002498345.1	[3]
UBA536	Permafrost soil (active layer), Nunavut, Canada	GCA_002496625.1	[3]
RRmetagenome_bin19	Polar desert soil, Wilkes Land, Antarctica	GCA_003176995.1	[4]

1. This study.

2. Pessi et al. (2022).

3. Parks et al. (2017), based on data originally published by Chauhan et al. (2014).

4. Ji et al. (2017).

### Genome annotation

We used anvi'o v7.0 (Eren et al. 2015) to predict gene calls with Prodigal v2.6.3 (Hyatt et al. 2010), identify rRNA genes and a set of 76 archaeal single-copy genes with HMMER v.3.3 (Eddy 2011), and compute genome completion and redundancy levels based on the presence of the 76 single-copy genes. We also employed anvi'o v7.0 to annotate the gene calls against the KOfam (Aramaki et al. 2020) and Pfam (Mistry et al. 2021) databases with HMMER v.3.3 (Eddy 2011) and the COG database (Galperin et al. 2021) with DIAMOND v0.9.14 (Buchfink et al. 2015). Additionally, we used BLASTP v2.10.1 (Camacho et al. 2009) to annotate the gene calls against the arCOG database (Makarova et al. 2015). Matches with scores below the precomputed family-specific thresholds (KOfam and Pfam), e-value > 10^–6^ (COG), or amino acid identity < 35% and coverage < 75% (arCOG) were discarded and, in case of multiple matches, the one with the lowest e-value was kept.

We used BLASTP v2.10.1 to compare the amino acid sequences of genes identified as *amoA*, *amoB*, *amoC*, or *amoX* to the RefSeq (O'Leary et al. 2016) and Swiss-Prot (The UniProt Consortium 2019) databases, and BLASTN v2.10.1 (Camacho et al. 2009) to compare *amoA* genes against the nt database and the curated *amoA* database of Alves et al. (2018). BLASTP v2.10.1 was also used to identify the putative *amoYZ* genes proposed recently by Hodgskiss et al. (2022, preprint). Functional enrichment analyses were carried out using anvi'o v7.0 (Eren et al. 2015) according to Shaiber et al. (2020). In brief, the occurrence of arCOG functions across genomes was summarized and logistic regression was then used to identify functions associated with a particular genus or genera. For this, we considered only the three most complete *Nitrososphaera*, *Ca*. Nitrosocosmicus, and *Ca*. Nitrosodeserticola genomes plus the representative genome of each *Ca*. Nitrosopolaris species.

### Phylogenomic and phylogenetic analyses

For phylogenomic analysis, we used a set of 59 archaeal single-copy genes that were present in at least 80% of the genomes. In addition to the 12 UBA10452MAGs, we retrieved from GenBank other 33 genomes belonging to the family Nitrososphaeraceae and the genome of *Nitrosopumilus maritimus* SCM1 to be used as an outgroup. We used anvi'o v7.0 (Eren et al. 2015) to recover the predicted amino acid sequence for each of the 59 genes, align them individually with MUSCLE v3.8.1551 (Edgar 2004), and generate a concatenated alignment. We then computed a maximum likelihood tree with IQ-TREE v2.1.4 employing the automatic model selection and 1000 bootstraps (Nguyen et al. 2015). Pairwise average nucleotide identity (ANI) values were computed with pyani v0.2.10 (Pritchard et al. 2016) and pairwise average amino acid identity (AAI) values with the AAI-Matrix tool (http://enve-omics.ce.gatech.edu/g-matrix).

Phylogenetic analysis of the *amoA* and 16S rRNA genes were done as described for the phylogenomic analysis (i.e. alignment with MUSCLE and tree building with IQ-TREE). Genes annotated as multicopper oxidase (PF07731, PF07732, COG2132, or arCOG03914) or nitrite reductase (K00368) were aligned with MAFFT v7.490 (Katoh and Standley 2013) alongside the sequences reported by Kerou et al. (2016), and a maximum likelihood tree was computed with IQ-TREE v2.1.4 (Nguyen et al. 2015) as described above.

### Abundance and geographic distribution

We employed read recruitment to compute the relative abundance of the UBA10452 lineage across the metagenomics datasets from which the MAGs were originally recovered. These datasets consisted of 10 Illumina NextSeq metagenomes from tundra soils in Rásttigáisá, Norway (this study); 69 Illumina NextSeq/NovaSeq metagenomes from tundra soils in Kilpisjärvi, Finland (Pessi et al. 2022); 13 Illumina HiSeq metagenomes from permafrost soils in Nunavut, Canada (Chauhan et al. 2014, Stackhouse et al. 2015); and three Illumina HiSeq metagenomes from polar desert soils in Wilkes Land, Antarctica (Ji et al. 2017). We used fasterq-dump v2.10.8 (https://github.com/ncbi/sra-tools) to retrieve the raw metagenomic data from the Sequence Read Archive (SRA). We then used CoverM v0.6.1 (https://github.com/wwood/CoverM) to map the reads to the MAGs with minimap v2.17 (Li 2016) and to compute relative abundances based on the proportion of reads recruited by the MAGs. In addition, we used IMNGS (Lagkouvardos et al. 2016) to further investigate the geographic distribution of the UBA10452 lineage. For this, we used the 16S rRNA gene sequence of the MAG RRmetagenome_bin19 as query to screen 422 877 amplicon sequencing datasets in SRA with UBLAST (Edgar 2010). We considered only matches with ≥ 99.0% similarity.

## Results

### Genomic characteristics of the UBA10452 lineage

**Figure 1.**
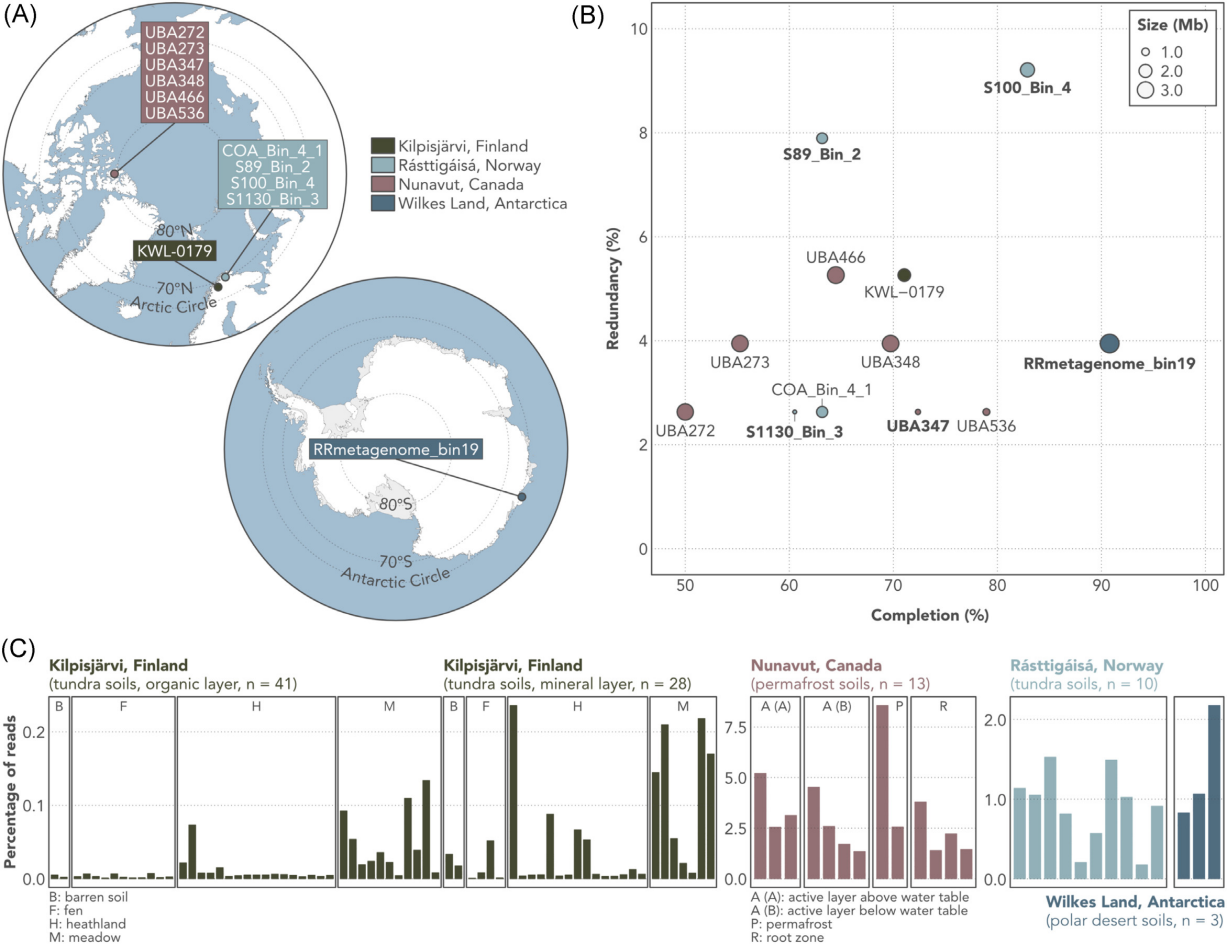
Geographic origin, assembly statistics, and abundance of MAGs assigned to the UBA10452 lineage (*Ca*. Nitrosopolaris). (A) Maps of the Arctic and Antarctic regions showing the geographic origin of the 12 UBA10452 MAGs. (B) Genome completion, redundancy, and size of the UBA10452 MAGs. Completion and redundancy levels were computed based on the presence of 76 single-copy genes. MAGs in bold include the 16S rRNA gene. (C) Proportion of metagenomic reads recruited by the UBA10452 MAGs across the four datasets from which they were originally recovered.

We applied a genome-resolved metagenomics approach to data obtained from tundra soils in Rásttigáisá, Norway, and recovered four MAGs assigned to the genus ‘UBA10452’, an uncultured lineage in the order Nitrososphaerales (‘terrestrial group I.1b’), phylum Thaumarchaeota. The UBA10452 lineage is currently represented by eight MAGs in GenBank in addition to the four MAGs obtained in the present study (Table 1 and Fig. 1A). Genome completion and redundancy estimated with anvi'o v7.0 (Eren et al. 2015) based on the presence of 76 single-copy genes range from 50.0% to 90.8% and 2.6% to 9.2%, respectively (Fig. 1B; Table S1, Supporting Information). The MAG RRmetagenome_bin19, with 90.8% completion, 3.9% redundancy, and a nearly complete (1462 bp) 16S rRNA gene, is a high-quality MAG according to the MIMAG standard (Bowers et al. 2017). The remaining 11 MAGs are of medium quality (≥ 50% complete and < 10% redundant), four of which also include the 16S rRNA gene. The genome size of UBA10452 MAGs ranges from 0.8 Mb (MAG S1130_Bin_3, 60.5% complete) to 4.0 Mb (MAG RRmetagenome_bin19, 90.8% complete). G+C content ranges from 38.1% to 41.5%.

### UBA10452 has a predominantly polar distribution

All 12 UBA10452 MAGs were obtained from cold (tundra, permafrost, and polar desert) soils (Table 1 and Fig. 1A). To gain insights into the ecology of the UBA10452 lineage, we used read recruitment to quantify the abundance of UBA10452 MAGs in the metagenomic datasets from which they were assembled. UBA10452 MAGs were most abundant in the dataset of permafrost from nutrient-poor (C: 1.0%, N: 0.1%) mineral cryosoils in Nunavut, Canadian Arctic, where they recruited up to 8.6% of the reads in each sample (Fig. 1C). On the other hand, UBA10452 MAGs were least abundant in the more nutrient-rich (C: 7.3%, N: 0.3%) tundra soils from Kilpisjärvi, Finland, where they were detected particularly in samples taken from the mineral layer of heathland and meadow soils.

In order to investigate further the geographic distribution of the UBA10452 lineage, we used IMNGS (Lagkouvardos et al. 2016) to screen 422 877 16S rRNA gene amplicon sequencing datasets in SRA. Sequences matching the 16S rRNA gene of UBA10452 MAGs (≥ 99.0% similarity) were found across 1281 datasets, mostly consisting of soil (*n* = 750), freshwater (*n* = 104), and rhizosphere samples (*n* = 100). Matched reads accounted for 6.0% of the total number of reads in these datasets (8.9 out of 149.1 million sequences). Of these, the overwhelming majority (8.7 million reads, 97.9%) come from Antarctic soil datasets, particularly from 149 sites in the vicinity of Davis Station, Princess Elizabeth Land (Bissett et al. 2016; Figure S1a, Supporting Information). The proportion of reads matching the UBA10452 lineage was above 50% of the archaeal 16S rRNA gene sequences in 70 of these sites and reached values as high as 88.8% (Figure S1b, Supporting Information).

### UBA10452 is a distinct lineage in the family Nitrososphaeraceae

**Figure 2.**
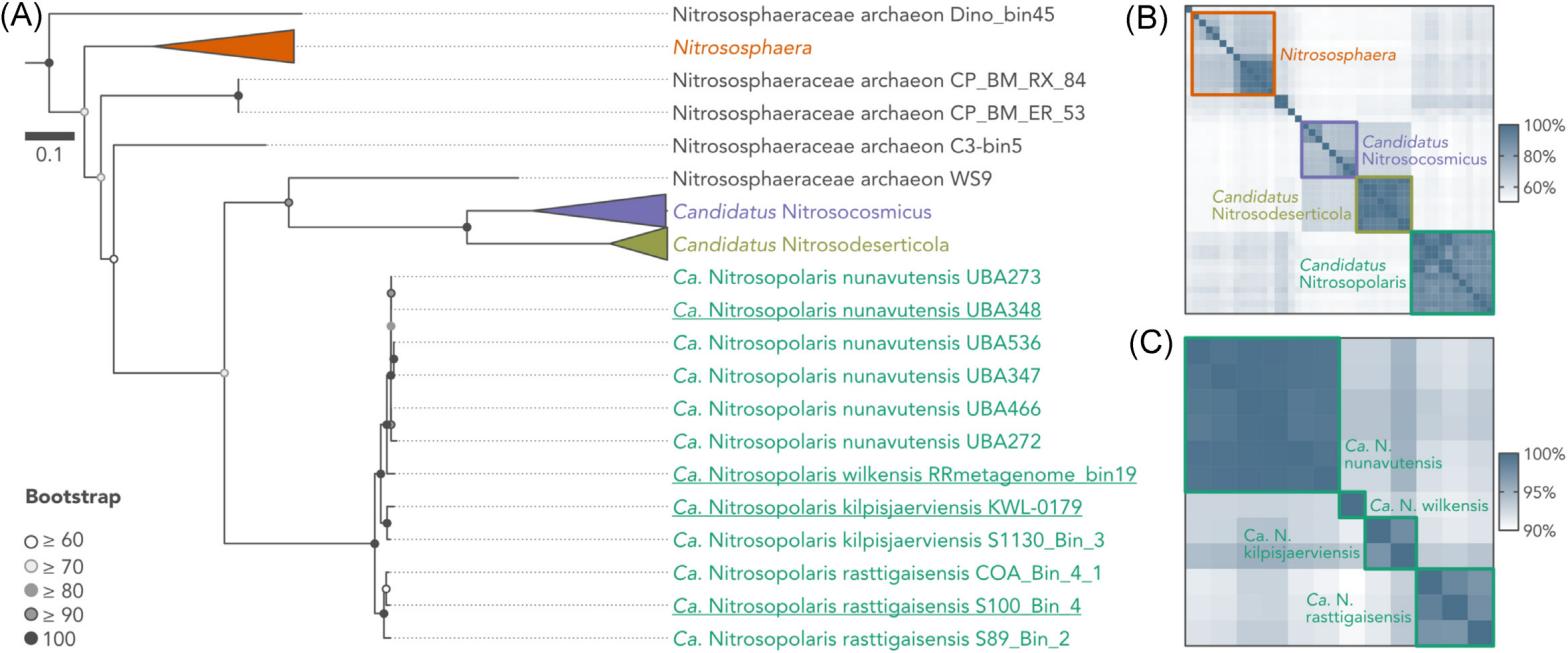
Phylogenomic analysis of the UBA10452 lineage (*Ca*. Nitrosopolaris). (A) Maximum likelihood tree based on 59 single-copy genes from 12 MAGs assigned to the UBA10452 lineage and 33 other Nitrososphaeraceae genomes available on GenBank. The tree was rooted with *N. maritimus* SCM1 (not shown). Representatives for the four proposed species are indicated in underscore. An uncollapsed version of the tree can be found in Figure S2 (Supporting Information). (B) Pairwise average AAI between Nitrososphaeraceae genomes and (C) ANI between UBA10452 MAGs. The boxes encompass the four described Nitrososphaeraceae genera (AAI threshold of 65%; panel B) and the four proposed species of *Ca*. Nitrosopolaris (ANI threshold of 95%–96%; panel C). Rows and columns are ordered from top to bottom and left to right, respectively, according to the top–bottom order of leaves in panel (A).

Phylogenomic analysis based on 59 single-copy genes placed the UBA10452 MAGs as a distinct lineage outside *Nitrososphaera*, *Ca*. Nitrosocosmicus, and *Ca*. Nitrosodeserticola, the three described genera in the family Nitrososphaeraceae (Stieglmeier et al. 2014, Lehtovirta-Morley et al. 2016, Hwang et al. 2021; Fig. 2A; Figure S2, Supporting Information). Separation of UBA10452 is also supported by AAI and 16S rRNA gene analyses. UBA10452 MAGs share 59.1% ± 1.9, 53.0% ± 1.1, and 53.8% ± 0.9 AAI with *Nitrososphaera*, *Ca*. Nitrosocosmicus, and *Ca*. Nitrosodeserticola, respectively (Fig. 2B), all of which are below the 65% AAI threshold commonly used to delineate microbial genera (Konstantinidis et al. 2017). At the 16S rRNA gene level, UBA10452 MAGs are 94.8% ± 1.2 and 95.4% ± 0.2 similar to *Nitrososphaera* and *Ca*. Nitrosocosmicus, respectively (Figure S3, Supporting Information). These values are in the limit of the 95% threshold for genus delineation proposed by Rosselló-Móra and Amann (2015), but are well-below the median 16S rRNA gene similarity observed between related genera across different microbial phyla (96.4%; Yarza et al. 2014). Comparison with *Ca*. Nitrosodeserticola was not possible due to the lack of a 16S rRNA gene sequence from this genus. Given that UBA10452 represents a clear, distinct lineage in the family Nitrososphaeraceae, we consider that UBA10452 should be recognized as a *Candidatus* genus and propose the name *Ca*. Nitrosopolaris.

Pairwise ANI values between *Ca*. Nitrosopolaris MAGs range from 90.9% to 99.9% (Fig. 2C). Based on either a 95% (Konstantinidis et al. 2017) or 96% ANI threshold (Ciufo et al. 2018), the 12 *Ca*. Nitrosopolaris MAGs can be separated into four distinct species (Fig. 2A; Figure S2, Supporting Information). A total of two of these, one comprising the six Canadian MAGs (Chauhan et al. 2014, Parks et al. 2017) and the other consisting solely of the Antarctic MAG (Ji et al. 2017), correspond to the two existing species in GTDB release 95 (‘UBA10452 sp002501855’ and ‘UBA10452 sp003176995’, respectively). Here, we suggest renaming these species as *Ca*. Nitrosopolaris nunavutensis and *Ca*. Nitrosopolaris wilkensis, respectively, according to the geographic origin of the MAGs. The Finnish MAG (Pessi et al. 2022) plus one of the Norwegian MAGs obtained in the present study (S1130_Bin_3) represent a novel species, for which we suggest the name *Ca*. Nitrosopolaris kilpisjaerviensis. Finally, the three remaining MAGs obtained in the present study (COA_Bin_4_1, S89_Bin_2, and S100_Bin_4) correspond to another novel species, which we propose to be named as *Ca*. Nitrosopolaris rasttigaisensis. However, the separation of *Ca*. Nitrosopolaris into four species is not supported by the analysis of the 16S rRNA gene (Figure S3, Supporting Information). The pairwise similarity between 16S rRNA gene sequences across the four ANI clusters range from 99.5% to 99.9%, which is above the 98.7%–99.0% threshold commonly used for species delineation (Stackebrandt and Ebers 2006, Kim et al. 2014).

### *Candidatus* Nitrosopolaris harbours genes for ammonia oxidation, CO_2_ fixation, and carbohydrate metabolism and transport

**Figure 3.**
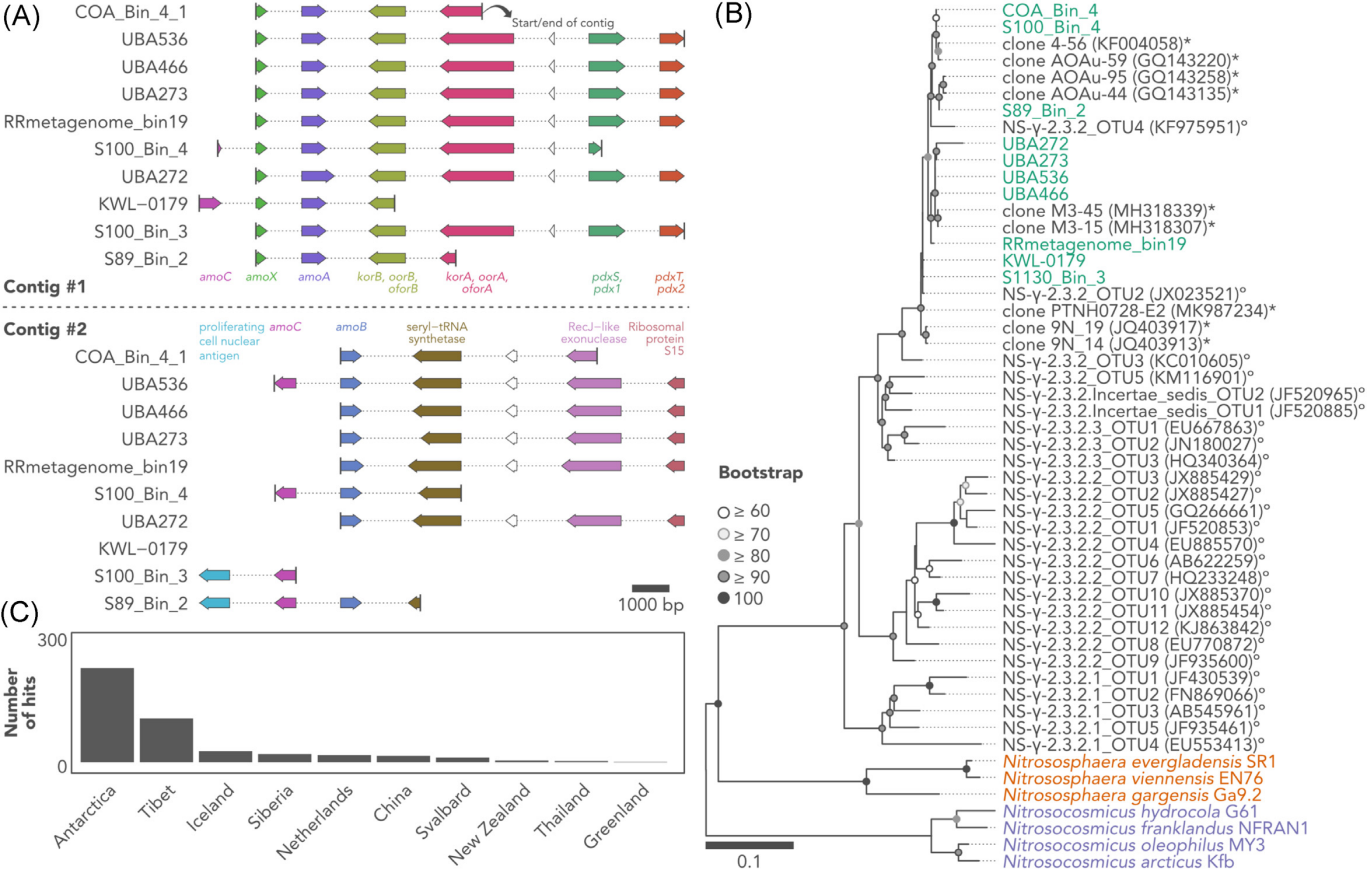
The *amo* genes of UBA10452 (*Ca*. Nitrosopolaris). (A) Representation of two contigs containing *amo* genes in MAGs assigned to the UBA10452 lineage. A total of two MAGs, which do not contain the *amoA* gene are omitted (UBA347 and UBA348). (B) Maximum likelihood tree of the *amoA* sequence of UBA10452MAGs and related sequences from GenBank (asterisks) and Alves et al. (2018) (circles). Bootstrap values < 60 are omitted. (C) Geographic origin of sequences from GenBank with ≥ 96.0% nucleotide similarity to the *amoA* sequence of UBA10452MAGs.

Annotation of protein-coding genes revealed that *Ca*. Nitrosopolaris harbours the *amoA*, *amoB*, *amoC*, and *amoX* genes encoding the enzyme Amo, which catalyzes the oxidation of ammonia to hydroxylamine (Fig. 3A; Figure S4 and Table S2, Supporting Information). Putative homologues of the *amoYZ* genes proposed by Hodgskiss et al. (2022, preprint) were found in all *Ca*. Nitrosopolaris MAGs, although with relatively low amino acid similarity for *amoZ* (mean ± sd: *amoY*, 82.6% ± 16.6%; *amoZ*, 32.3% ± 1.4%). As for other AOA, homologues of the *hao* gene were not found in the *Ca*. Nitrosopolaris MAGs. In AOB, this gene encodes the enzyme hydroxylamine dehydrogenase (Hao), which takes part in the oxidation of hydroxylamine to nitrite, a mechanism that remains unknown in AOA (Lehtovirta-Morley 2018). A proposed mechanism of hydroxylamine oxidation in AOA is via a copper-containing nitrite reductase (NirK) encoded by the *nirK* gene, which has been detected in most *Ca*. Nitrosopolaris MAGs together with other related multicopper oxidases (Figure S5, Supporting Information). *Ca*. Nitrosopolaris also encodes an ammonium transporter of the Amt family involved in the uptake of extracellular ammonium. Moreover, *Ca*. Nitrosopolaris harbours urease (*ureABC*) and urea transporter (*utp*) genes, indicating the ability to generate ammonia from urea. In contrast to *Nitrososphaera gargensis* (Spang et al. 2012), we did not detect the *cynS* gene encoding the enzyme cyanate hydratase involved in the production of ammonia from cyanate.

The *amoABCX* genes in all *Ca*. Nitrosopolaris MAGs are distributed across two separate contigs (Fig. 3A). One of the contigs contains the *amoC*, *amoX*, and *amoA* genes; however, the *amoC* gene is truncated and found in only two MAGs. In some MAGs, the other contig contains a second, full-length copy of the *amoC* gene followed by *amoB*. Not all MAGs contain all *amoABCX* genes. However, considering that the MAGs present varying levels of completion (Fig. 1B; Table S1, Supporting Information) and since the localization of the genes corresponds to start or end of contigs (Fig. 3A), it is likely that missing genes are an artifact of truncated assemblies rather than due to gene loss. With few exceptions, putative *amoYZ* genes (Hodgskiss et al. 2022, preprint) were located adjacent to each other but in a different contig from the other *amo* genes. In some MAGs, this contig contained the ammonium transporter *amt* gene at 9–10 genes away from the *amoYZ* cluster.

The *amoA* gene of *Ca*. Nitrosopolaris has a length of 651 bp and belongs to the NS-γ-2.3.2 cluster of Alves et al. (2018; Fig. 3B). One exception is the MAG UBA272, which contains a longer *amoA* gene (873 bp) with a long insert of ambiguous base calls, most likely an artifact from assembling and/or scaffolding. Sequences belonging to the NS-γ-2.3.2 cluster are found majorly in acidic soils (Alves et al. 2018). Moreover, analysis of sequences from GenBank showed that the *amoA* gene of *Ca*. Nitrosopolaris is related (≥ 96% nucleotide similarity) to sequences recovered mostly from cold environments, i.e. the Artic, Antarctica, and alpine regions such as the Tibetan Plateau (Fig. 3C). Among these, the *amoA* sequences of *Ca*. Nitrosopolaris MAGs are most closely related (98.9%–99.7% nucleotide similarity) to uncultured sequences from Antarctic soil (MH318339 and MH318307), grassland soil in Iceland (JQ403917 and JQ403913), and the Tibetan Plateau (GQ143258, GQ143220, GQ143135, KF004058, and MK987234; Daebeler et al. 2012, Xie et al. 2014, Wang et al. 2019, Zhang et al. 2019; Fig. 3B).

Similarly to other AOA, *Ca*. Nitrosopolaris harbours genes for the hydroxypropionate–hydroxybutyrate pathway of CO_2_ fixation, complexes I–V of the electron transfer chain, the citric acid cycle, and gluconeogenesis (Figure S4 and Table S2, Supporting Information). Like other AOA, the gene content of *Ca*. Nitrosopolaris indicates a potential for mixotrophic metabolism, with multiple copies of genes encoding proteins involved in carbohydrate metabolism and transport such as glucose/sorbosone dehydrogenases, permeases of the major facilitator superfamily (MFS), and pyruvate oxidases. In contrast to *Nitrososphaera*, we did not detect genes involved in the assembly of pili, flagellar apparatus (archaellum), and chemotaxis.

### *Candidatus* Nitrosopolaris MAGs are enriched in genes involved in DNA replication and repair

**Figure 4.**
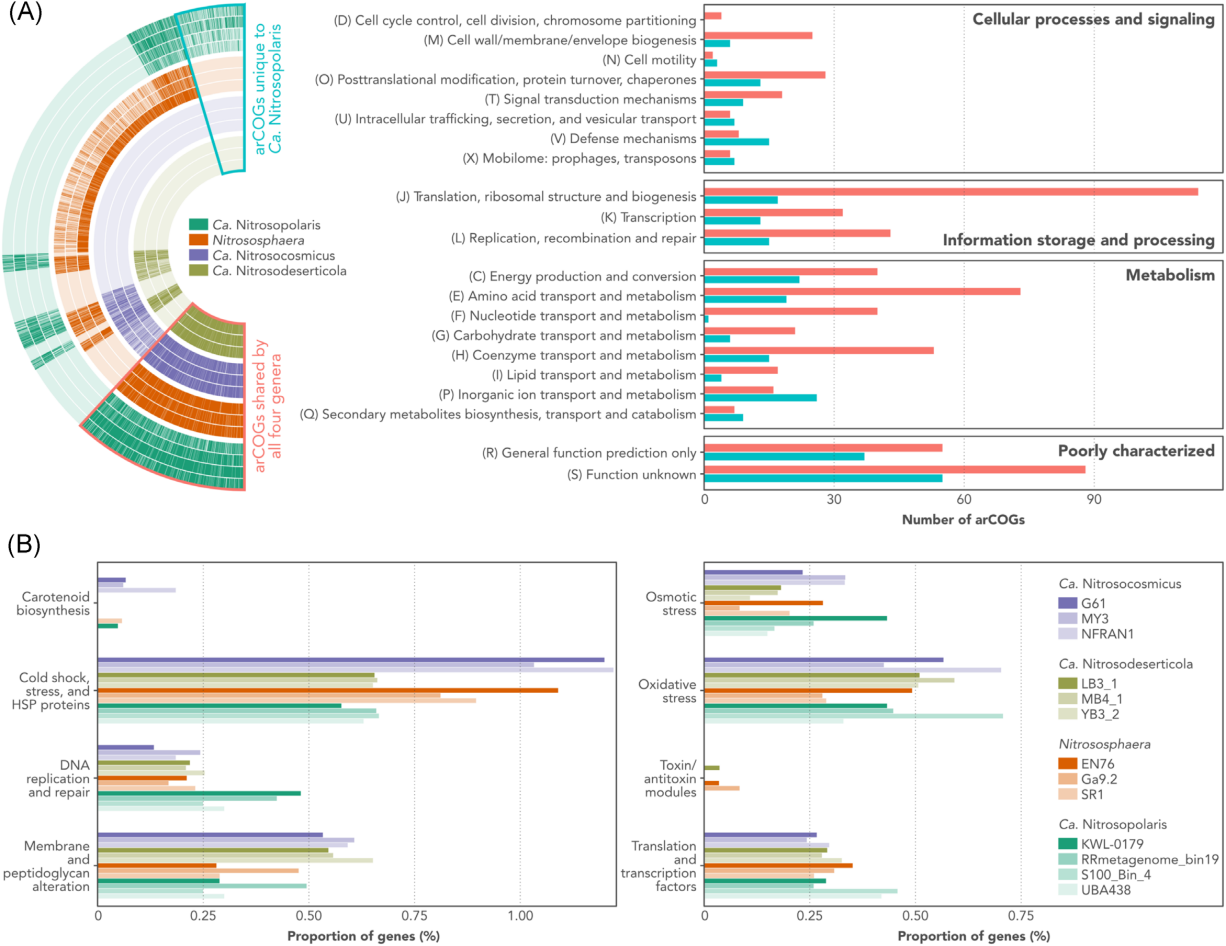
Comparative genomics of the UBA10452 lineage (*Ca*. Nitrosopolaris) and other members of the family Nitrososphaeraceae. (A) Functional enrichment analysis showing arCOG functions shared by all Nitrososphaeraceae genera and other functions unique to *Ca*. Nitrosopolaris. More detail on the arCOG functions unique to *Ca*. Nitrosopolaris can be found in Table S3 (Supporting Information). (B) Distribution of genes with known or predicted roles in cold adaptation and growth. Number of genes is shown as a proportion of the total number of genes in each genome. More detail on the genes found in *Ca*. Nitrosopolaris can be found in Table S4 (Supporting Information).

To investigate possible mechanisms underlying the distribution of *Ca*. Nitrosopolaris, we carried out a functional enrichment analysis covering the four Nitrososphaeraceae genera. In total, the 13 MAGs used in the analysis encoded 3999 different arCOG functions (Fig. 4A). Of these, 948 functions were shared among all four genera and 368 were unique to *Ca*. Nitrosopolaris. Of the arCOG functions shared by all four genera, most belonged to the classes translation, ribosomal structure, and biogenesis (*n* = 114), function unknown (*n* = 88), and amino acid transport and metabolism (*n* = 73). On the other hand, arCOG functions unique to *Ca*. Nitrosopolaris belonged mostly to the classes function unknown (*n* = 59), general function prediction only (*n* = 52), and inorganic ion transport and metabolism (*n* = 33). Among these are several types of hydrolases, lipoproteins, phospholipases, and ABC transporters including ones for iron, maltose, phosphate, amino acids, and nucleosides (Table S3, Supporting Information).

In addition to the genome-wide functional enrichment analysis, we also looked more specifically for genes with known or predicted roles in cold adaptation and growth (Raymond-Bouchard et al. 2018). When comparing the genomic repertoire of *Ca*. Nitrosopolaris to the other Nitrososphaeraceae genera, the former was found to harbour a higher number of genes involved in DNA replication and repair (Fig. 4B). More specifically, *Ca*. Nitrosopolaris MAGs encode multiple copies of the enzymes RecA ATPases and RecA/RadA recombinases (Table S4, Supporting Information). Surprisingly, genes related to cold shock response were less abundant in *Ca*. Nitrosopolaris compared to *Nitrososphaera* and *Ca*. Nitrosocosmicus (Fig. 4B), although several copies of molecular chaperones (DnaK, GrpE, and IbpA) and universal stress proteins (UspA) were identified (Table S4, Supporting Information). In addition to these, *Ca*. Nitrosopolaris also harbours several copies of other genes encoding proteins related to cold adaptation and growth (Fig. 4B), including proteins involved in membrane and peptidoglycan alteration (glycosyltransferases), osmotic stress (sodium–hydrogen antiporters and sodium–proline symporters), oxidative stress (periredoxins and thioredoxin reductases), and translation/transcription (DNA/RNA helicases and transcription factors; Table S4, Supporting Information).

## Discussion

Genome-resolved metagenomics has revolutionized our knowledge of archaeal diversity by giving us access to the genome of uncultured microorganisms at an unprecedented rate (Tahon et al. 2021). In a recent metagenomic investigation of tundra soils in northern Finland (Pessi et al. 2022), we have manually binned and curated a MAG belonging to the genus ‘UBA10452’, an uncultured and largely uncharacterized lineage in the order Nitrososphaerales (‘terrestrial group I.1b’) of the phylum Thaumarchaeota. Here, we binned four other UBA10452 MAGs from tundra soils in Rásttigáisá, Norway, and characterized the phylogeny, metabolic potential, and biogeography of this lineage. Our results indicate that the UBA10452 lineage consists of putative AOA with a geographic distribution mostly restricted to cold ecosystems, particularly the polar regions. We suggest the recognition of UBA10452 as a *Candidatus* genus, for which we propose the name *Ca*. Nitrosopolaris (*nitrosus*: Latin adjective meaning nitrous; *polaris*: Latin adjective meaning of or pertaining to the poles).

The findings from our polyphasic analysis consisting of phylogenomic, AAI, and 16S rRNA gene analyses support the placement of *Ca*. Nitrosopolaris outside *Nitrososphaera*, *Ca*. Nitrosocosmicus, and *Ca*. Nitrosodeserticola in the family Nitrososphaeraceae, as previously suggested (Rinke et al. 2021). Our results further indicate that the 12 *Ca*. Nitrosopolaris MAGs represent four different species based on a 95%–96% ANI threshold (Konstantinidis et al. 2017, Ciufo et al. 2018). In addition to the two current species in GTDB release 95 (Parks et al. 2018, 2020), the inclusion of the four MAGs obtained in the present study resulted in the identification of two novel species. It is important to note that the separation of *Ca*. Nitrosopolaris into four species based on ANI values is not readily supported by the analysis of 16S rRNA gene sequences, which are ≥ 99.5% similar across the four species. Although a 98.7%–99.0% threshold is commonly used (Stackebrandt and Ebers 2006), species delineation based solely on the 16S rRNA gene can be problematic given that microorganisms belonging to different species can share identical 16S rRNA gene sequences (Kim et al. 2014, Schloss 2021). An example of it is *Ca*. Nitrosocosmicus arcticus and *Ca*. Nitrosocosmicus oleophilus, two species of AOA which share an identical 16S rRNA gene sequence despite having divergent genomes with only 83.0% ANI (Alves et al. 2019). It thus appears reasonable to conclude that the 12 *Ca*. Nitrosopolaris MAGs indeed represent four different species as suggested by the ANI analysis. If cultured representatives become available in the future, phenotypic and ecophysiological characterization of these isolates could help resolve the taxonomy of *Ca*. Nitrosopolaris.

*Ca*. Nitrosopolaris harbours the complete set of *amoABCX* genes responsible for chemolithotrophic growth via ammonia oxidation (Lehtovirta-Morley 2018). In addition, putative homologues of the recently proposed *amoYZ* genes were found, although with low confidence for *amoZ*. These genes have been linked to two potential additional Amo subunits in *Nitrososphaera viennensis* and other AOA, but further studies are needed to confirm this association (Hodgskiss et al. 2022, preprint). The putative ammonia oxidation capability of *Ca*. Nitrosopolaris is supported by its close phylogenetic relationship to *Nitrososphaera* and *Ca*. Nitrosocosmicus, two genera which have been demonstrated to grow by oxidizing ammonia (Stieglmeier et al. 2014, Lehtovirta-Morley et al. 2016). In addition, the presence of several genes involved in carbohydrate and amino acid transport and metabolism suggest that *Ca*. Nitrosopolaris, like other AOA, might be able to grow mixotrophically using organic compounds as alternative energy and/or C sources (Mußmann et al. 2011, Pester et al. 2011). Nevertheless, although *in silico* analyses provide valuable predictions, metabolic capabilities inferred by genomic annotation need to be confirmed based on the analysis of isolated/enriched cultures or with the help of other indirect methods such as stable isotope probing (SIP; Gadkari et al. 2020).

In addition to the geographical origin of the MAGs, large-scale screening of 16S rRNA gene and *amoA* sequences from SRA and GenBank indicate that *Ca*. Nitrosopolaris is restricted to soils in the cold biosphere. The soils from which the *Ca*. Nitrosopolaris MAGs have been recovered are typical of polar and alpine environments, being characterized by low pH (4.8–5.1), carbon (C; 1.0%–7.3%), and N (0.1%–0.3%) content (Stackhouse et al. 2015, Ji et al. 2017, Pessi et al. 2022). Furthermore, the abundance profile of *Ca*. Nitrosopolaris observed in this study, which was characterized by a higher abundance in mineral cryosoil permafrost and polar desert soils compared to vegetated tundra soils, indicates that *Ca*. Nitrosopolaris is particularly adapted to the highly oligotrophic conditions found in some of the most extreme environments in the cryosphere. The discovery of *Ca*. Nitrosopolaris complements the list of microbial taxa that appear to be adapted to life in cold environments, such as the mat-forming cyanobacteria *Phormidesmis priestleyi* (Komárek et al. 2009) and *Shackeltoniella antarctica* (Strunecky et al. 2020) and the sea-ice bacteria *Polaribacter* and *Psychrobacter* (Bowman 2013).

Investigation of the genome of *Ca*. Nitrosopolaris provided insights on possible adaptations to cold and oligotrophic environments. For instance, *Ca*. Nitrosopolaris harbour multiple copies of several genes that have been implicated in tolerance to cold, such as genes encoding proteins involved in DNA replication and repair, molecular chaperones, DNA/RNA helicases, and universal stress proteins (Raymond-Bouchard et al. 2018). Interestingly, *Ca*. Nitrosopolaris appears to be enriched in copies of the RecA enzyme compared to other members of the Nitrososphaeraceae. RecA plays a key role in DNA repair, which is an important mechanism for survival in polar environments where DNA is frequently damaged due to freezing and UV radiation (Cavicchioli 2006). In addition to the possible adaptive mechanisms of *Ca*. Nitrosopolaris, it has been suggested that the environmental characteristics of polar soils favour AOA in general (Alves et al. 2013, Siljanen et al. 2019). The ecological success of AOA in oligotrophic and acidic soils has been traditionally linked to the higher affinity of their ammonia oxidation machinery compared to their bacterial counterparts (Martens-Habbena et al. 2009, Kerou et al. 2016), although a recent study has shown that high affinity for ammonia is not common to all AOA (Jung et al. 2022). Furthermore, the hydroxypropionate–hydroxybutylate pathway of CO_2_ fixation encoded by *Ca*. Nitrosopolaris and other AOA appears to be more energy efficient than the Calvin cycle employed by AOB (Könneke et al. 2014). However, these traits are shared between *Ca*. Nitrosopolaris and other AOA and, thus do not readily explain the apparent ecological success of *Ca*. Nitrosopolaris in cold environments. Indeed, mechanisms of cold adaptation are evolutionary and functionally complex and involve many features that cannot be observed by metagenomics alone (e.g. gene regulation and membrane modifications; Cavicchioli 2006). Structural, transcriptomics, and proteomics analysis of cultured isolates could help shed further light on possible adaptations to cold in *Ca*. Nitrosopolaris.

In addition to possible mechanisms of adaptation to polar environments, we hypothesize that the distribution of *Ca*. Nitrosopolaris could be, to some extent, related to historical factors. Interestingly, the four proposed *Ca*. Nitrosopolaris species form coherent biogeographic clusters: *Ca*. N. nunavutensis, comprising MAGs obtained from permafrost soils in Nunavut, Canada; *Ca*. N. wilkensis, corresponding to one MAG from polar desert soils in Wilkes Land, Antarctica; and *Ca*. N. kilpisjaerviensis and *Ca*. N. rasttigaisensis, comprising MAGs obtained from mineral tundra soils in two relatively close regions in northern Fennoscandia (Kilpisjärvi and Rásttigáisá, respectively). A recent molecular dating study has suggested that the origin of the AOA clade group I.1b (order Nitrososphaerales) coincides with severe glaciation events that happened during the Neoproterozoic (Yang et al. 2021). If these estimates are accurate, it would imply that *Ca*. Nitrosopolaris and all other lineages in group I.1b share a common ancestor that appeared when the global climate was characterized by subzero temperatures, having likely evolved at glacial refugia, such as nunataks or regions with geothermal activity.

Due to low temperatures throughout the year, polar soils store a large amount of organic matter and thus have served as important carbon sinks. At present, polar soils are considered minor yet significant sources of N_2_O (Voigt et al. 2020) but, if warming trends continue at the levels observed currently, polar soils might become major contributors to the global N_2_O budget. For instance, the AOA *Ca*. N. arcticus isolated from Arctic soil has an ammonia oxidation optimum at temperatures well above those found *in situ* (Alves et al. 2019). Given that both the direct and indirect roles of AOA in the cycling of N_2_O in polar soils remain largely undetermined, a better understanding of polar microbial communities is paramount to model current and future N_2_O fluxes from this biome.

## Supplementary Material

xtac019_Supplemental_Files

## Data Availability

Genomic assemblies can be found in GenBank/ENA under the accession numbers listed in Table 1. MAGs generated in this study have been submitted to ENA (BioProject PRJEB49283). All the code used can be found in https://github.com/ArcticMicrobialEcology/candidatus-nitrosopolaris.
