# Supplementary material for: Candidatus Nitrosopolaris, a genus of putative ammonia-oxidizing archaea with a polar/alpine distribution
Source: FEMS Microbes. 2022 Jun 24;3:xtac019. doi: 10.1093/femsmc/xtac019 (PMC10117904; doi:10.1093/femsmc/xtac019)
Supplement: xtac019_Supplemental_Files [file xtac019_supplemental_files.zip › AOA_v3_suppl_figs_1.docx]

*FEMS Microbes*

*Candidatus* Nitrosopolaris, a genus of putative ammonia-oxidizing archaea with a polar/alpine distribution

Igor S. Pessi^1,2^, Aino Rutanen^1^, and Jenni Hultman^1,2,3*^

^1^Department of Microbiology, University of Helsinki, Helsinki, Finland

^2^Helsinki Institute of Sustainability Science (HELSUS), Helsinki, Finland

^3^Natural Resources Institute Finland (LUKE), Helsinki, Finland

^*^Corresponding author: [jenni.hultman@helsinki.fi](mailto:jenni.hultman@helsinki.fi)

# Supplementary Figures


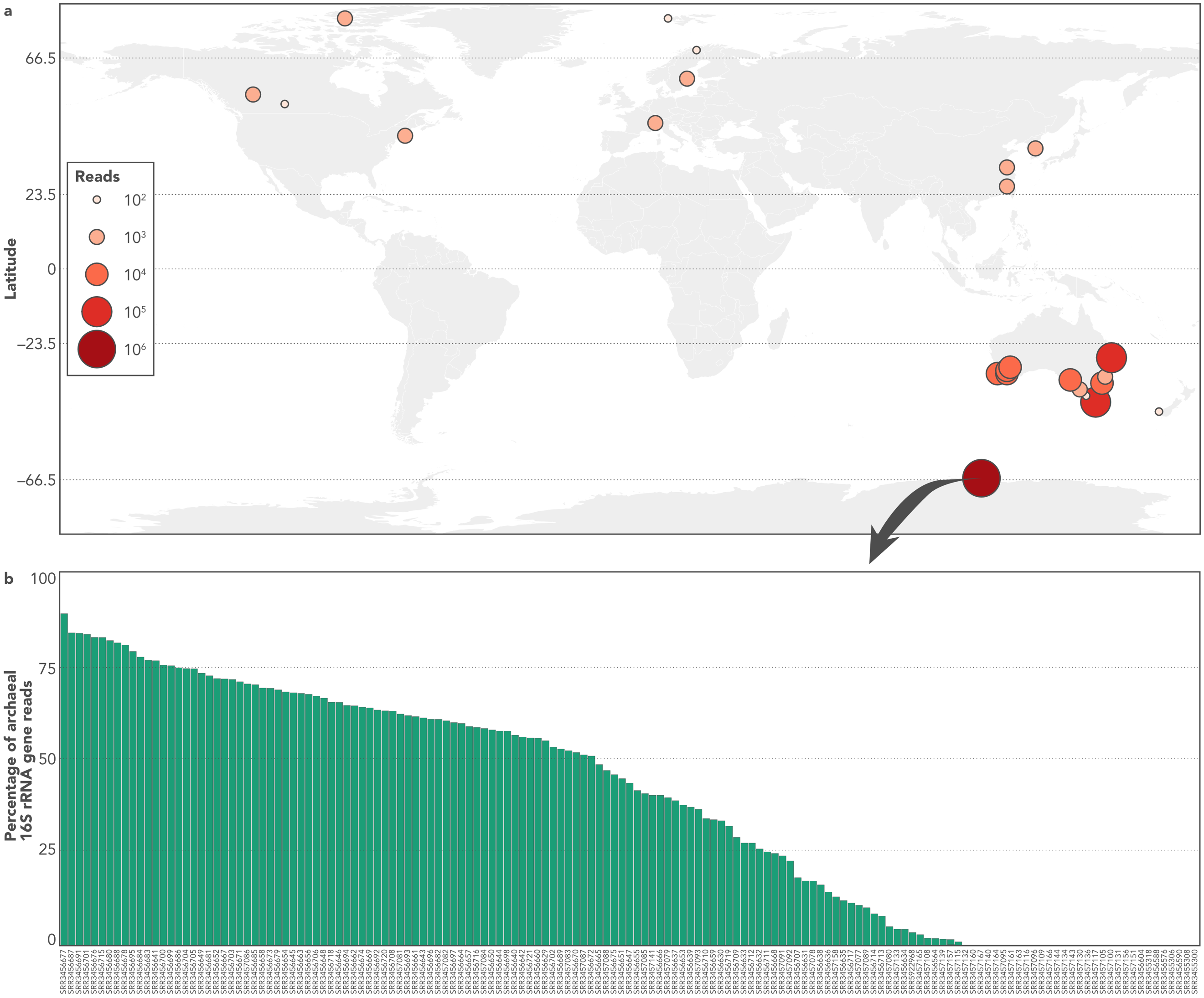


**Suppl. Figure S1. Geographic distribution of the UBA10452 lineage (*Candidatus* Nitrosopolaris). a)** Distribution of *Ca*. Nitrosopolaris based on the screening of 422,877 16S rRNA gene amplicon sequencing datasets in the Sequence Read Archive (SRA). Datasets with few matches (< 0.1% or < 100 reads) are not shown. **b)** Abundance of *Ca*. Nitrosopolaris across 149 16S rRNA gene amplicon sequencing datasets from soils in the vicinity of Davis Station, Princess Elizabeth Land, Antarctica (BioProject PRJNA317932). Relative abundances were computed as the proportion of reads matching the sequence of *Ca*. Nitrosopolaris in each sample. Abundances represent the percentage of *Ca*. Nitrosopolaris reads relative to archaeal 16S rRNA gene reads obtained with archaea-specific primers.


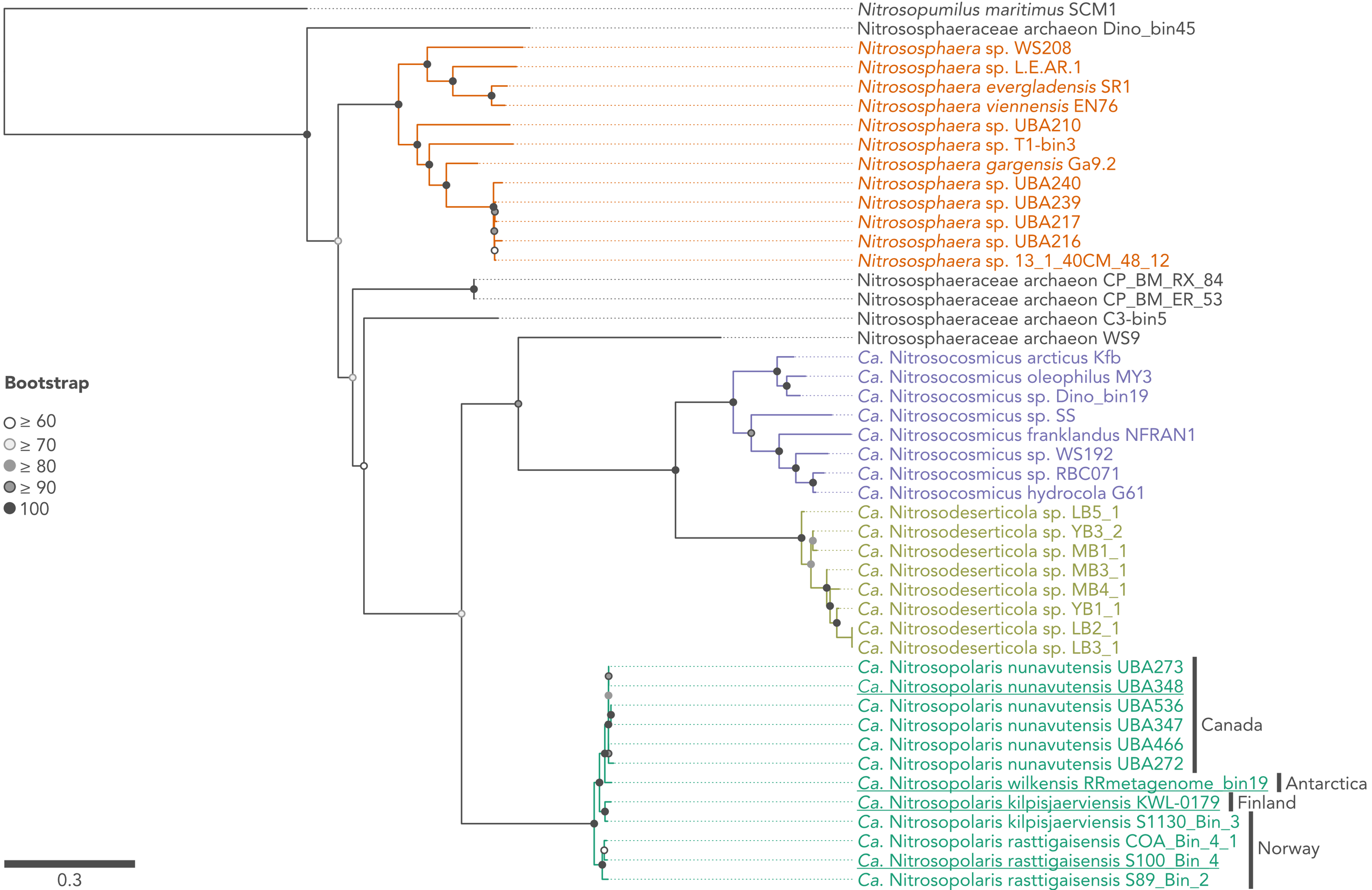


**Suppl. Figure S2. Phylogenomic analysis of the UBA10452 lineage (*Candidatus* Nitrosopolaris).** Maximum likelihood tree based on 59 single-copy genes from 12 metagenome-assembled genomes (MAGs) assigned to the UBA10452 lineage and 33 other Nitrososphaeraceae genomes available on GenBank. *Nitrosopumilus maritimus* SCM1 was used for rooting the tree. Representatives for the four proposed species are indicated in underscore. This is an uncollapsed version of the tree in **Fig. 2a**.


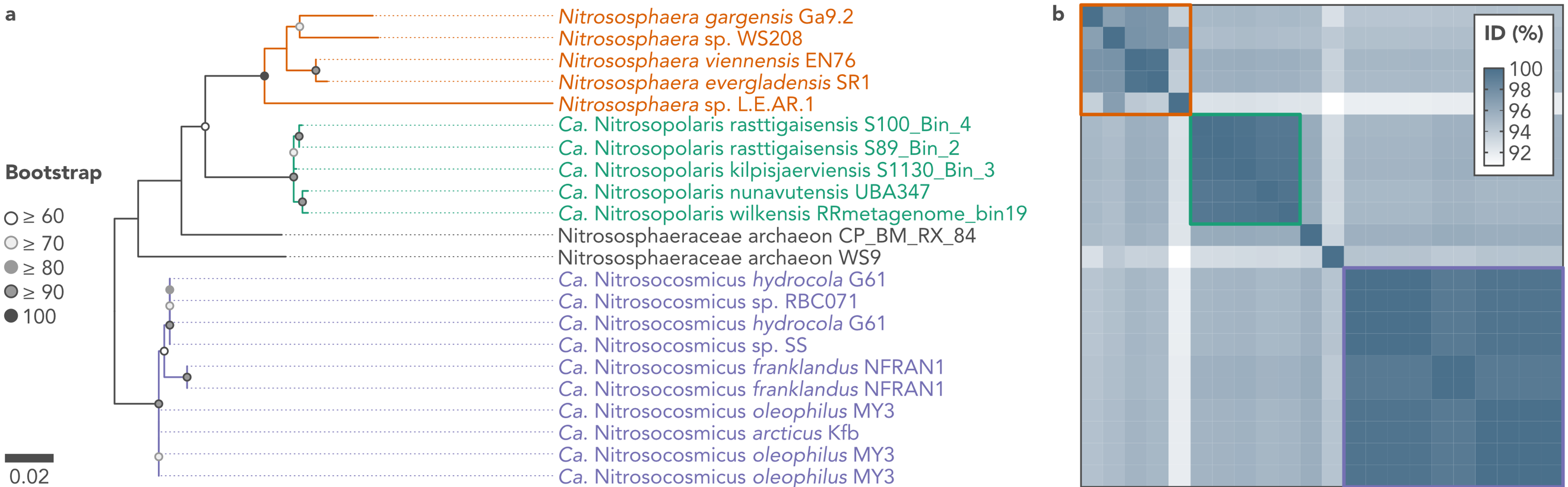


**Suppl. Figure S3. The 16S rRNA gene of UBA10452 (*Candidatus* Nitrosopolaris). a)** Phylogenetic analysis of the 16S rRNA gene sequence of five metagenome-assembled genomes (MAGs) assigned to the UBA10452 lineage and other Nitrososphaeraceae genomes available on GenBank. Maximum likelihood tree rooted with *Nitrosopumilus maritimus* SCM1 (not shown). Bootstrap values < 60 are omitted. **b)** Pairwise similarity between 16S rRNA gene sequences from panel a. Note that some genomes contain multiple copies of the 16S rRNA gene.


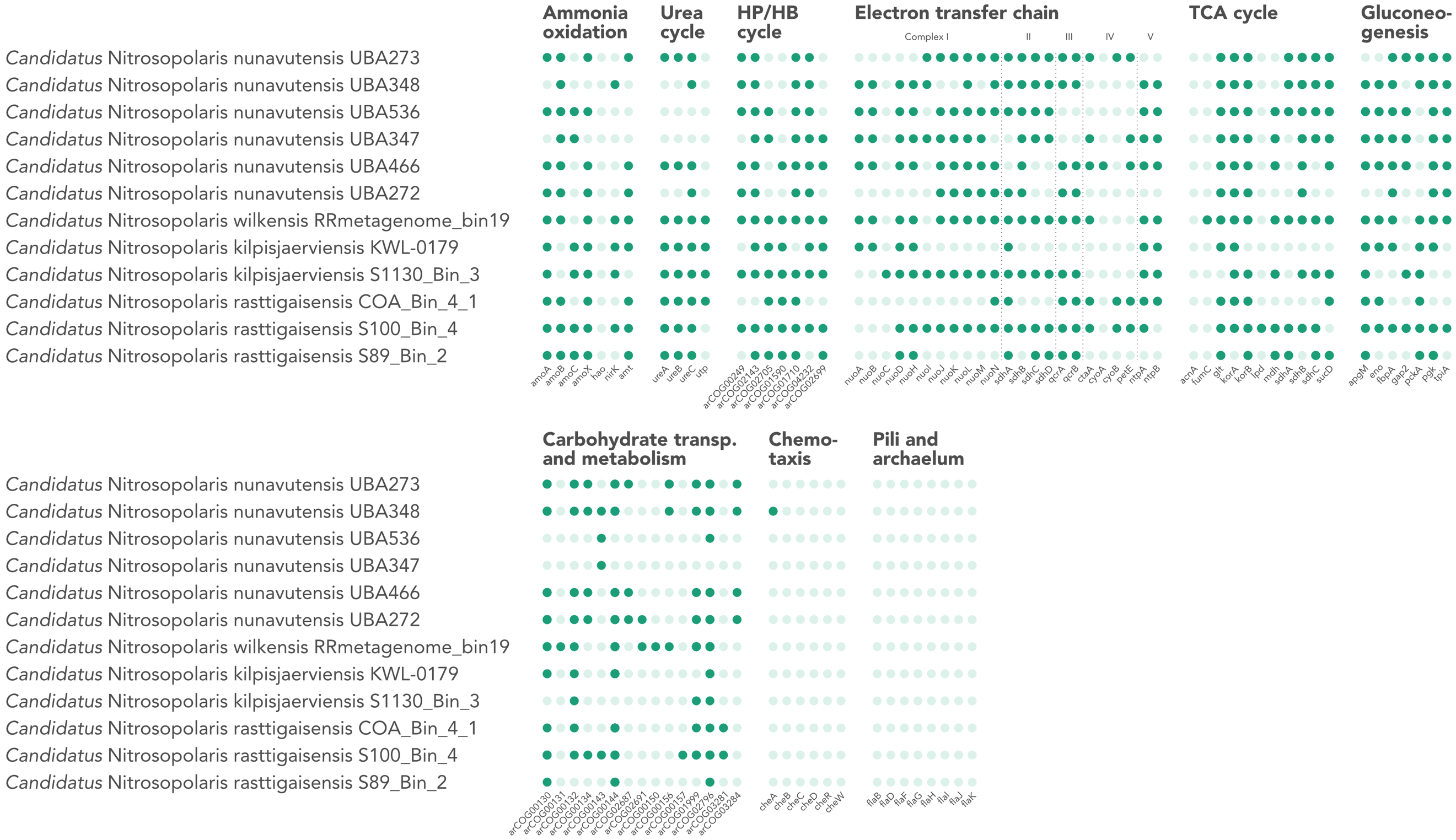


**Suppl. Figure S4. Metabolic potential of the UBA10452 lineage (*Candidatus* Nitrosopolaris).** Metabolic potential was estimated based on the presence of key genes involved in selected pathways. Detailed information about the genes can be found in **Suppl. Table S2**.


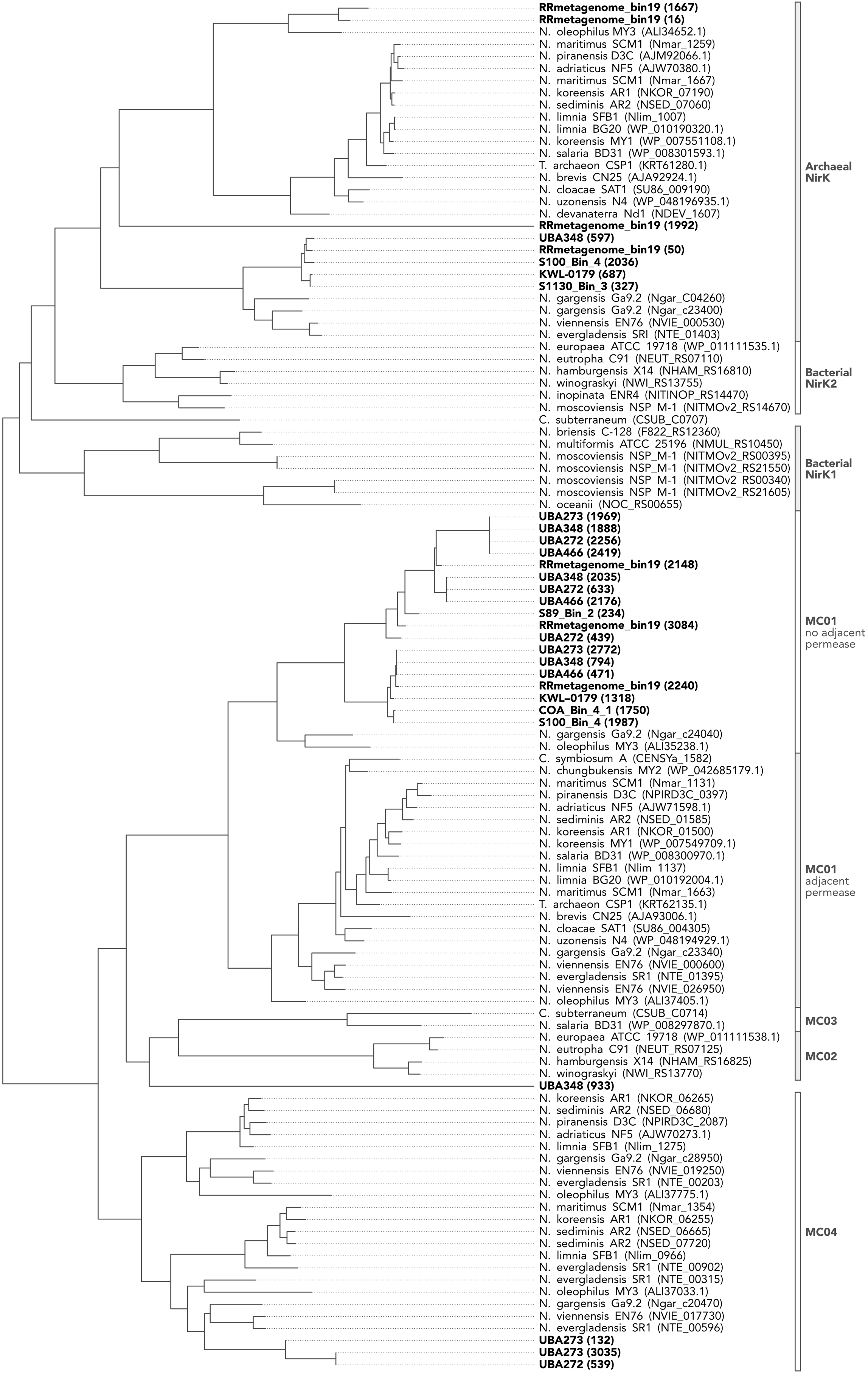


**(Previous page) Suppl. Figure S5. Phylogenetic analysis of putative NirK sequences from metagenome-assembled genomes (MAGs) belonging to the UBA10452 lineage (*Candidatus* Nitrosopolaris).** Sequences from the UBA10452 are shown in bold and respective gene calls are given inside parenthesis. Other sequences were retrieved from Kerou *et al*. (2016). All nodes have bootstrap support ≥ 80%.
