# Supplementary material for: Candidatus Nitrosopolaris, a genus of putative ammonia-oxidizing archaea with a polar/alpine distribution
Source: FEMS Microbes. 2022 Jun 24;3:xtac019. doi: 10.1093/femsmc/xtac019 (PMC10117904; doi:10.1093/femsmc/xtac019)
Supplement: xtac019_Supplemental_Files [file xtac019_supplemental_files.zip › Reviews_and_Author_Response.docx]

**Reviews of manuscript FEMSMC-2021-061**

**Reviewer 1 - Anne Daebeler (Biology Centre CAS, Soil and Water Research Infrastructure)**

**Recommendation: Minor revision**

Pessi and colleagues are presenting an analysis of novel and previously published AOA MAGs that informs on an AOA genus without cultured representatives. Next to phylogenomic and biogeographical analyses, they have performed comparative genomcis to shed light on the potential ecophysiology, phylogeny and global occurrence of the microbes represented in this genus. Their bioinformatic analysis has led them to identify four new species and justifies the Candidatus naming of the genus. The data presented is of high quality and the manuscript is written very well. I only have a few minor suggestions.

- At then end of the abstract and also in the discussion the authors speculate that limited dispersal capabilities due to a probable lack of flagellum (or rather archaellum!) and the chemotaxis inventory are a likely reason for the restricted global occurrence patterns of the genus Ca. Nitrosopolaris. I agree, that this genus seems to be preferentially present in polar and alpine regions, but I do not think that the lack of motility is a likely reason for this pattern. That is because, I do not think that the absence of an archaellum and connected chemotaxis sensors limit the dispersal per se of a soil microorganism on a global scale. In fact, if thermophilic AOA like N. islandicus and N. cavascurensis are dispersed between Icelandic and Italian hot springs, as obvious from their very high genomic similarity but distant occurance, then I would argue that Ca. Nitrosopolaris species are subject to similar dispersal forces, possibly by wind and ocean currents. My guess would be, that like Ca. Nitrosocaldus, Ca. Nitrospolaris species are dispersed just as readily as other microbes, but are very specialized AOA with narrow environmental niches which restrict their colonisation ability.

- line 34 and in discussion: Please take into account the recent findings by Jung et al (doi: 10.1038/s41396-021-01064-z) of also lower apparent affinity for ammonia in AOA. This new evidence shows that within AOA there are genera with a similarly low apparent affinity for their substrate as some AOB.

- Please indicate in the main text that your recent work cited throughout the manuscript has not yet undergone peer-review. I suggest referring to this reference like so: (Pessi et al, 2021; preprint).

- line 78: change to “... genomic, potential metabolic, and...”

- Fig.2b: Which ANI cut-off do the coloured lines around the depicted genera represent? Its difficult to guess from the legend. Please include in figure caption.

- I would be interested to know if the authors were able to detect the presence of hydrogenases or other genes connected to potential alternative energy metabolism in the analysed MAGs? Also, are nirK and multicopper oxidases present in these MAGs and if yes, into which types can they be classified according to Kerou et al 2016 (doi: 10.1073/pnas.1601212113)?

- line 330: The authors could include Schloss 2021 (doi: https://doi.org/10.1128/mSphere.00191-21) here for a more recent reference.

- line 341ff: There is no hard proof that any member of the Nitrososphaera can not oxidize ammonia. Please correct.

**Reviewer 2 - Man-Young Jung (Chungbuk National University, Department of Microbiology)**

**Recommendation: Reject**

The manuscript offers a comprehensive description of a metagenome-assembled genomes (MAGs) of ammonia-oxidizing Thaumarchaeota from the novel group of cluster 1.1b as based on 16S rRNA and genomic phylogeny. While the paper is well written and offers basic information regarding this analysis of the genomes from acidic polar and alpine soils, it is essentially descriptive information. Even with the genome sequence, there is no full accounting for how or why this MAG should be notably different or important relative to other ammonia-oxidizing thaumarchaeal genera. There is no comprehensive description with a specific analysis of the main properties, cold adaptation of the novel AOA genus. All of the studies are based on genomic and transcriptomic characterization. This means that the potential properties could be suggested but not verified. Phrases should be revised in this aspect. Plus, there are many aspects in the materials and methods, results, and discussion that are unclear. Also, if the authors want to focus on the dominance of the new genus, they should show their results more in the importance and abundance in the acidic polar and alpine soils to make it more straightforward for the reader.

Other comments

1) Over-interpretation of data about the new AOA genus. The mere presence of genome sequences of AOA MAGs does not necessarily mean the new genus. Without AOA in culture, the new genus of AOA cannot be emphasized in the title.

2) Re-organization of crucial story. The key story is metagenome-enabled metatranscriptome analysis. The authors need to stick to the critical data showing (a) the relative abundance of AOA-like genes in the total communities and confirm to use qPCR; (b) discuss the possible importance of these different AOA, and explain why the novel genus of AOA is unique when compared to other AOA clads regarding cold adaptation mechanism.

3) Need to explain sequencing details. Because author also used recruitment metagenome sequencing from a database which was analyzed by different sequencing methods.

4) The introduction is somehow like a comprehensive review. So please keep focused on the key story of the MS.

Specific comments

L33-36: please check the reference Jung et al.,2022, that always and all AOA affinities do not have a higher affinity than AOB.

L40: Why Thermoproteota?

L65-66: profer reference is needed here.

L76: it is still Thaumarchaeota.

L81: it is needed information on how the genome was analyzed and how to prepare, collect, analyze the genome sample.

Figure 2: there are not many isolated AOA genomes; why was it not included? And are those LB2_1, LB3_1, and so on, belong to the genus Nitrosocosmicus?

L212-213 in Figure 3: It would not be matched because of the low 16s rRNA gene ratio in the metagenome.

L222: 8.6% ratio is so high. Please recheck it.

L230, 234-235: It seems like to unusually high ratio. Is it this like high? If so, why the reason?

L244: I don’t know why all separated into two contigs? There is no detection in other contigs?

L250-253: it is not clear whether it is about ammonia or ammonium?

L309-312: There is the threshold for defining genus level (for ANI and AAI, also for 16S rRNA gene), as far as I know correctly. Please recheck it.

Table S1. It is not possible to see the table at a glance.

**Author's Response to Decision Letter for (FEMSMC-2021-061)**

Dear Editor Dr. Jana Jass,

We thank you and the two anonymous reviewers for the valuable comments on our manuscript “Candidatus Nitrosopolaris, a genus of putative ammonia-oxidizing archaea with a polar/alpine distribution”. As detailed below, we have taken these comments into thorough consideration and have made several changes to the manuscript. However, we have to disagree with several points raised by Reviewer 2, some of which do not concern specific issues such as methodology/interpretation of results but more subjective issues such as how we have decided to frame our study, things which were not criticized by Reviewer 1. More specifically:

Reviewer 2 states that “The mere presence of genome sequences of AOA MAGs does not necessarily mean the new genus”. We strongly disagree with this point of view and have detailed in the point-by-point reply below how our proposal of a novel Candidatus genus based on high-quality metagenome-assembled genomes is in line with the current standards of microbiological taxonomy (see e.g. Konstantinidis et al. 2017). We also would like to point out that Reviewer 1 has also manifested support to the creation of a novel Candidatus genus.

Reviewer 2 has misunderstood the methodology used in our study and, in consequence, the main story of our manuscript. Contrary to what the reviewer has written (“The key story is metagenome-enabled metatranscriptome analysis”), we have used metagenomics to investigate the diversity of microorganisms in tundra soils and our main story revolves around the description of a novel genus of AOA. For some reason, the reviewer is under the impression that we have used metatranscriptomics, which is not mentioned anywhere in the manuscript. Thus, the reviewer’s suggestion “The authors need to stick to the critical data showing the relative abundance of AOA-like genes in the total communities and confirm to use qPCR” completely misses the point of our manuscript.

Some of the comments made by Reviewer 2 were very cryptic. In one comment, the reviewer has stated “proper reference is needed”. We have indeed provided a reference for the sentence in question and because we cannot guess what the reviewer means by “proper reference”, we were not able to act upon it. In another comment, the reviewer has written “L212-213 in Figure 3: It would not be matched because of the low 16s rRNA gene ratio in the metagenome”. We were not able to figure out what would not be matched to what, and did not understand the reference to the 16S rRNA gene which has nothing to do with what the data presented in the figure.

We have, nevertheless, completely revised our manuscript with the aim of clarifying potential issues that might lead to misunderstanding of our story, as was the case of Reviewer 2. We hope you will find that our manuscript has been greatly improved and would now be suitable for publication on FEMS Microbes.

On behalf of the co-authors,

Jenni Hultman, PhD

Docent (Adjunct Professor) in Microbiology and Academy Research Fellow

Department of Microbiology, University of Helsinki, Finland

Reviewer: 1

Pessi and colleagues are presenting an analysis of novel and previously published AOA MAGs that informs on an AOA genus without cultured representatives. Next to phylogenomic and biogeographical analyses, they have performed comparative genomcis to shed light on the potential ecophysiology, phylogeny and global occurrence of the microbes represented in this genus. Their bioinformatic analysis has led them to identify four new species and justifies the Candidatus naming of the genus. The data presented is of high quality and the manuscript is written very well. I only have a few minor suggestions.

We thank the reviewer for their interest in our work. Please find our point-by-point reply below.

At then end of the abstract and also in the discussion the authors speculate that limited dispersal capabilities due to a probable lack of flagellum (or rather archaellum!) and the chemotaxis inventory are a likely reason for the restricted global occurrence patterns of the genus Ca. Nitrosopolaris. I agree, that this genus seems to be preferentially present in polar and alpine regions, but I do not think that the lack of motility is a likely reason for this pattern. That is because, I do not think that the absence of an archaellum and connected chemotaxis sensors limit the dispersal per se of a soil microorganism on a global scale. In fact, if thermophilic AOA like N. islandicus and N. cavascurensis are dispersed between Icelandic and Italian hot springs, as obvious from their very high genomic similarity but distant occurance, then I would argue that Ca. Nitrosopolaris species are subject to similar dispersal forces, possibly by wind and ocean currents. My guess would be, that like Ca. Nitrosocaldus, Ca. Nitrospolaris species are dispersed just as readily as other microbes, but are very specialized AOA with narrow environmental niches which restrict their colonisation ability.

We thank the reviewer for these insights. Indeed, upon careful consideration, we agree that lack of motility would be a weak explanation for the distributional patterns of Ca. Nitrosopolaris. We have thus modified the text accordingly:

Based on the phylogenetic, biogeographical, and ecological characteristics of 12 UBA10452 MAGs, which include a high-quality MAG (90.8% complete, 3.9% redundant) with a nearly complete 16S rRNA gene, we propose a novel Candidatus genus, Ca. Nitrosopolaris, with four species representing clear biogeographical/habitat clusters.

Abstract, lines 18–22

A recent molecular dating study has suggested that the origin of the AOA clade group I.1b (order Nitrososphaerales) coincides with severe glaciation events that happened during the Neoproterozoic (Yang et al., 2021). If these estimates are accurate, it would imply that Ca. Nitrosopolaris and all other lineages in group I.1b share a common ancestor that appeared when the global climate was characterized by sub-zero temperatures, having likely evolved at glacial refugia such as nunataks or regions with geothermal activity.

Discussion, lines 441–446

line 34 and in discussion: Please take into account the recent findings by Jung et al (doi: 10.1038/s41396-021-01064-z) of also lower apparent affinity for ammonia in AOA. This new evidence shows that within AOA there are genera with a similarly low apparent affinity for their substrate as some AOB.

We thank the reviewer for bringing our attention to this important study. We have added:

Among the reasons for their ecological success is an enzymatic machinery with higher affinity for ammonia and a more efficient CO2 fixation pathway than their bacterial counterparts (Martens-Habbena et al., 2009; Könneke et al., 2014; Kerou et al., 2016). However, high ammonia affinity is not a common trait to all AOA, with some strains displaying a low substrate affinity that is comparable to that of non-oligotrophic AOB (Kits et al., 2017; Jung et al., 2022).

Introduction, lines 31–36

The ecological success of AOA in oligotrophic and acidic soils has been traditionally linked to the higher affinity of their ammonia oxidation machinery compared to their bacterial counterparts (Martens-Habbena et al., 2009; Kerou et al., 2016), although a recent study has shown that high affinity for ammonia is not common to all AOA (Jung et al., 2022).

Discussion, lines 421–425

Please indicate in the main text that your recent work cited throughout the manuscript has not yet undergone peer-review. I suggest referring to this reference like so: (Pessi et al, 2021; preprint).

We have incorporated the reviewer’s suggestion.

line 78: change to “... genomic, potential metabolic, and...”

The sentence has been revised:

Here, we i) report four novel UBA10452 MAGs obtained from tundra soils in Rásttigáisá, Norway; ii) characterize the genomic properties, metabolic potential, phylogeny, and biogeography of the UBA10452 lineage; and iii) propose the creation of a new Candidatus genus, Ca. Nitrosopolaris.

Introduction, lines 72–75

Also in the discussion:

Here, we binned four other UBA10452 MAGs from tundra soils in Rásttigáisá, Norway, and characterized the phylogeny, metabolic potential, and biogeography of this lineage.

Discussion, lines 360–362

Fig.2b: Which ANI cut-off do the coloured lines around the depicted genera represent? Its difficult to guess from the legend. Please include in figure caption.

We thank the reviewer for this suggestion. In fact, we have modified the figure substantially to, among other things, show AAI values instead of ANI for comparing the different Nitrososphaeraceae genera as AAI is better suited for distantly related genomes (panel B). In panel C, we now show the ANI values only between the different Ca. Nitrosopolaris MAGs. The coloured lines in panels B and C demarcate the different genera (65% AAI) and the different Ca. Nitrosopolaris species (95–96% ANI), respectively. This information has been added to the figure caption as suggested by the reviewer:

Figure 2. Phylogenomic analysis of the UBA10452 lineage (Candidatus Nitrosopolaris). a) Maximum likelihood tree based on 59 single-copy genes from 12 metagenome-assembled genomes (MAGs) assigned to the UBA10452 lineage and 33 other Nitrososphaeraceae genomes available on GenBank. The tree was rooted with Nitrosopumilus maritimus SCM1 (not shown). Representatives for the four proposed species are indicated in underscore. An uncollapsed and bootstrapped version of the tree can be found in Suppl. Fig. S2. b) Pairwise average amino acid identity (AAI) between Nitrososphaeraceae genomes and c) average nucleotide identity (ANI) between UBA10452 MAGs. The boxes encompass the four described Nitrososphaeraceae genera (AAI threshold of 65%; panel b) and the four proposed species of Candidatus Nitrosopolaris (ANI threshold of 95–96%; panel c). Rows and columns are ordered from top to bottom and left to right, respectively, according to the top-bottom order of leaves in panel a.

I would be interested to know if the authors were able to detect the presence of hydrogenases or other genes connected to potential alternative energy metabolism in the analysed MAGs? Also, are nirK and multicopper oxidases present in these MAGs and if yes, into which types can they be classified according to Kerou et al 2016 (doi: 10.1073/pnas.1601212113)?

We thank the reviewer for this suggestion. We have indeed found nirK and nirK-like sequences and have analysed them in relationship with the sequences from Kerou et al. 2016. We did not, however, detected hydrogenases in the Ca. Nitrospolaris MAGs. Information about the nirK sequences has been added to Suppl. Fig. S5:

Suppl. Figure S5. Phylogenetic analysis of putative NirK sequences from metagenome-assembled genomes (MAGs) belonging to the UBA10452 lineage (Candidatus Nitrosopolaris). Sequences from the UBA10452 are shown in bold and respective gene calls are given inside parenthesis. Other sequences were retrieved from Kerou et al. (2016).

A sentence has also been added to the results section:

As for other AOA, homologues of the hao gene were not found in the Ca. Nitrosopolaris MAGs. In AOB, this gene encodes the enzyme hydroxylamine dehydrogenase (Hao) which takes part in the oxidation of hydroxylamine to nitrite, a mechanism that remains unknown in AOA (Lehtovirta-Morley, 2018). A proposed mechanism of hydroxylamine oxidation in AOA is via a copper-containing nitrite reductase (NirK) encoded by the nirK gene, which has been detected in most Ca. Nitrosopolaris MAGs as well as other related multicopper oxidases (Suppl. Fig. S4).

Results, lines 271–276

line 330: The authors could include Schloss 2021 (doi: https://doi.org/10.1128/mSphere.00191-21) here for a more recent reference.

We thank the reviewer for the suggestion. We have added the suggested reference and have also added a reference for a 16S rRNA gene threshold for species delineation:

Although a 98.7–99.0% threshold is commonly used (Stackebrandt and Ebers, 2006), species delineation based solely on the 16S rRNA gene can be problematic given that microorganisms belonging to different species can share identical 16S rRNA gene sequences (Kim et al., 2014; Schloss, 2021).

Discussion, lines 376–379

line 341ff: There is no hard proof that any member of the Nitrososphaera can not oxidize ammonia. Please correct.

Thank you for this comment. The sentence has been deleted, and the part about putative mixotrophic growth has been merged with another sentence further down the paragraph:

In addition, the presence of several genes involved in carbohydrate and amino acid transport and metabolism suggest that Ca. Nitrosopolaris, like other AOA, might be able to grow mixotrophically using organic compounds as alternative energy and/or C sources (Mussmann et al., 2011; Pester et al., 2011).

Discussion, lines 394–397

Reviewer: 2

The manuscript offers a comprehensive description of a metagenome-assembled genomes (MAGs) of ammonia-oxidizing Thaumarchaeota from the novel group of cluster 1.1b as based on 16S rRNA and genomic phylogeny. While the paper is well written and offers basic information regarding this analysis of the genomes from acidic polar and alpine soils, it is essentially descriptive information. Even with the genome sequence, there is no full accounting for how or why this MAG should be notably different or important relative to other ammonia-oxidizing thaumarchaeal genera. There is no comprehensive description with a specific analysis of the main properties, cold adaptation of the novel AOA genus. All of the studies are based on genomic and transcriptomic characterization. This means that the potential properties could be suggested but not verified. Phrases should be revised in this aspect. Plus, there are many aspects in the materials and methods, results, and discussion that are unclear. Also, if the authors want to focus on the dominance of the new genus, they should show their results more in the importance and abundance in the acidic polar and alpine soils to make it more straightforward for the reader.

We thank the reviewer for these valuable comments. We have improved the manuscript by including a comparative analysis with other genera in the family Nitrososphaeraceae and have provided more details on putative genes associated with cold adaptation, including detailed information about the genes given in supplementary tables. We hope the reviewer will find that these additions contribute to a more comprehensive description of the unique features of Candidatus Nitrosopolaris. Moreover, we have thoroughly revised the text to make sure that we are indeed talking about potential properties based on in silico annotation rather than verified activities. The methods section has been thoroughly revised to include a full description of how the MAGs were obtained instead of just referring to previous studies from our group. Finally, we have reorganised the abstract, introduction, results, and discussion sections to make our story more straightforward to the reader. For example, paragraphs 1 and 2 in the discussion were merged and shortened considerably, and the description of the environments from which the MAGs originate has been expanded. Below we provide a point-by-point reply to the more detailed comments.

Over-interpretation of data about the new AOA genus. The mere presence of genome sequences of AOA MAGs does not necessarily mean the new genus. Without AOA in culture, the new genus of AOA cannot be emphasized in the title.

We have to disagree with the reviewer on this point. Indeed, the taxonomic challenges represented by uncultured taxa have been widely discussed by microbiologists but no consensus has so far been attained (see e.g. Konstantinidis et al. 2017; Murray et al. 2020). To quote Konstantinidis et al. 2017: “We are convinced that a classification of yet uncultured taxa based on binning of metagenomic data or single-cell genomics can be as stable and reliable as that established for cultured Bacteria and Archaea”. In this sense, the modern scientific literature is abundant with studies describing novel Candidatus taxa based on environmental sequences which, in our opinion, provide a much needed taxonomic framework considering how much of the archaeal and bacterial diversity remains uncultured (see e.g. Ji et al. 2017; Hwang et al. 2021; Luo et al. 2021; Rinke et al. 2021). This is particularly true for AOA, which are notoriously difficult to isolate.

We have analysed in our study 12 MAGs, some of which are almost complete and include the 16S rRNA gene. Our analyses – including 16S rRNA gene phylogeny, multi-gene phylogenomics, and ecological and biogeographical characterizations – provide robust evidence that these MAGs represent a distinct genus and warrants the creation of a novel Candidatus genus, as recognized by Reviewer 1. Indeed, our work fulfils the minimum standard proposed by Konstantinidis et al. 2017 regarding the description of uncultured taxa: i) analysis of a complete or almost complete genome sequence (> 80% completeness; < 5% contamination), including ANI/AAI comparison with close relatives and in silico prediction of metabolic traits; ii) ecological description of the habitat(s) in which the organism is found and its abundance, which has been improved in the current manuscript; and iii) phylogenetic analysis of the complete 16S rRNA gene. Thus, we stand by our proposal of a novel Candidatus genus and leave this issue to the Editor’s discretion to be evaluated according to the policy of FEMS Microbes regarding the description of uncultured taxa.

Re-organization of crucial story. The key story is metagenome-enabled metatranscriptome analysis. The authors need to stick to the critical data showing (a) the relative abundance of AOA-like genes in the total communities and confirm to use qPCR; (b) discuss the possible importance of these different AOA, and explain why the novel genus of AOA is unique when compared to other AOA clads regarding cold adaptation mechanism.

We believe that the reviewer has misunderstood the key story of our manuscript. First, we have not used in our study anything that would resemble what the reviewer calls “metagenome-enabled metatranscriptome analysis”. We believe that the reviewer is mistaking metagenomics, the tool used in our study, with metatranscriptomics, a related technique that is based on the sequencing of environmental RNA and is often used to quantify microbial processes in the environment. The focus of our study, contrary to what the reviewer has suggested, was not to quantify “AOA-like genes” and thus the reviewer’s proposal of adding qPCR analyses is not justified in our opinion.

We have, however, improved the manuscript considerably to better emphasize the uniqueness of Ca. Nitrospolaris as suggested by the reviewer. For example, a new analysis of functional comparative genomics has been added:

Ca. Nitrosopolaris MAGs are enriched in genes involved in DNA replication and repair

To investigate possible mechanisms underlying the distribution of Ca. Nitrosopolaris, we carried out a functional enrichment analysis covering the four Nitrososphaeraceae genera. In total, the 13 MAGs used in the analysis encoded 3,999 different arCOG functions (Fig. 4a). Of these, 948 functions were shared among all four genera and 368 were unique to Ca. Nitrosopolaris. Of the arCOG functions shared by all four genera, most belonged to the arCOG classes translation, ribosomal structure, and biogenesis (n = 114), function unknown (n = 88), and amino acid transport and metabolism (n = 73). On the other hand, arCOG functions unique to Ca. Nitrosopolaris belonged mostly to the arCOG classes function unknown (n = 59), general function prediction only (n = 52), and inorganic ion transport and metabolism (n = 33). Among these arCOG functions are several types of hydrolases, lipoproteins, phospholipases, and ABC transporters including ones for iron, maltose, phosphate, amino acids, and nucleosides (Suppl. Table S3).

Results, lines 317–330

Moreover, the paragraph about putative cold adaptation genes has been improved:

In addition to the genome-wide functional enrichment analysis, we also looked more specifically for genes with known or predicted roles in cold adaptation and growth (Raymond-Bouchard et al., 2018). When comparing the genomic repertoire of Ca. Nitrosopolaris to the other Nitrososphaeraceae genera, the former was found to harbour a higher number of genes involved in DNA replication and repair (Fig. 4b). More specifically, Ca. Nitrosopolaris MAGs encode multiple copies of the enzymes RecA ATPases and RecA/RadA recombinases (Suppl. Table S4). Surprisingly, genes related to cold shock response were less abundant in Ca. Nitrosopolaris compared to Nitrososphaera and Ca. Nitrosocosmicus (Fig. 4b), although several copies of molecular chaperones (DnaK, GrpE, and IbpA) and universal stress proteins (UspA) were identified (Suppl. Table S4). In addition to these, Ca. Nitrosopolaris also harbours several copies of other genes encoding proteins related to cold adaptation and growth (Fig. 4b), including proteins involved in membrane and peptidoglycan alteration (glycosyltransferases), osmotic stress (sodium-hydrogen antiporters and sodium-proline symporters), oxidative stress (periredoxins and thioredoxin reductases), and translation/transcription (DNA/RNA helicases and transcription factors) (Suppl. Table S4).

Results, lines 331–345

The results concerning this section are presented in the newly added Figure 4, and detailed information about the genes is given in Suppl. Table S3 and S4.

Need to explain sequencing details. Because author also used recruitment metagenome sequencing from a database which was analyzed by different sequencing methods.

We have added the sequencing method of each of the metagenome analysed:

We employed read recruitment to compute the relative abundance of the UBA10452 lineage across the metagenomics datasets from which the MAGs were originally recovered. These datasets consisted of 10 Illumina NextSeq metagenomes from tundra soils in Rásttigáisá, Norway (this study); 69 Illumina NextSeq/NovaSeq metagenomes from tundra soils in Kilpisjärvi, Finland (Pessi et al., 2022, pre-print); 13 Illumina HiSeq metagenomes from permafrost soils in Nunavut, Canada (Chauhan et al., 2014; Stackhouse et al., 2015); and three Illumina HiSeq metagenomes from polar desert soils in Wilkes Land, Antarctica (Ji et al., 2017).

Methods, lines 163–169

We believe that this do not affect our results given that Illumina HiSeq, NextSeq and NovaSeq are highly comparable as they share similar sequencing chemistry. The main difference between these methods is the total throughput; for this reason, the results from the read recruitment analysis were normalised based on library size by computing the percentage of matched reads rather than absolute values.

The introduction is somehow like a comprehensive review. So please keep focused on the key story of the MS.

We thank the reviewer for this suggestion. We have modified the introduction by removing some parts that could be seen as unnecessary, e.g.:

Nitrification – the oxidation of ammonia to nitrite and further oxidation to nitrate – is a crucial part of the nitrogen (N) cycle providing a link between reduced and oxidized forms of N. The first step of nitrification, ammonia oxidation, is carried out mainly by aerobic chemolithoautotrophic microorganisms that grow by coupling the energy obtained from the oxidation of ammonia with carbon dioxide (CO2) fixation (Lehtovirta-Morley, 2018).

Introduction, lines 24–28

AOA are notoriously difficult to cultivate and so far only three genera have been formally described based on axenic cultures: Nitrosopumilus (Qin et al., 2017) and Nitrosarchaeum (Jung et al., 2018) in the order Nitrosopumilales (“marine group I.1a”) and Nitrososphaera (Stieglmeier et al., 2014) in the order Nitrososphaerales (“terrestrial group I.1b”).

Introduction, lines 51–54

We have also switched the order of paragraphs 2 and 3, as we believe that it helps bringing more attention to one of the key point of our story, i.e., ammonia-oxidizing archaea in polar soils.

L33-36: please check the reference Jung et al.,2022, that always and all AOA affinities do not have a higher affinity than AOB.

We thank the reviewer for bringing our attention to this important study, which was also suggested by Reviewer 1. We have added:

Among the reasons for their ecological success is an enzymatic machinery with higher affinity for ammonia and a more efficient CO2 fixation pathway than their bacterial counterparts (Martens-Habbena et al., 2009; Könneke et al., 2014; Kerou et al., 2016). However, high ammonia affinity is not a common trait to all AOA, with some strains displaying a low substrate affinity that is comparable to that of non-oligotrophic AOB (Kits et al., 2017; Jung et al., 2022).

Introduction, lines 31–36

The ecological success of AOA in oligotrophic and acidic soils has been traditionally linked to the higher affinity of their ammonia oxidation machinery compared to their bacterial counterparts (Martens-Habbena et al., 2009; Kerou et al., 2016), although a recent study has shown that high affinity for ammonia is not common to all AOA (Jung et al., 2022).

Discussion, lines 421–425

L40: Why Thermoproteota?

Thermoproteota was the name chosen by Rinke et al. 2021 in their re-evaluation of the TACK (Thaumarchaeota–Aigarchaeota–Crenarchaeota–Korarchaeota) superphylum. To quote Rinke et al. 2021: “The TACK superphylum was reclassified as a single phylum based on rank normalization and its robust monophyly, for which we propose to use the effectively published name Thermoproteota based on the earliest validly described class for any TACK phylum, the Thermoprotei. Note that the phylum name could alternatively have been derived from the earliest genus name in the TACK superphylum, Sulfolobus; however, this genus is currently a member of the class Thermoprotei”. The sentence in question has nevertheless been removed to make the introduction more streamlined as suggested above.

L65-66: profer reference is needed here.

It is not clear what the reviewer means by “proper reference”. We have cited a comprehensive review on N2O production by AOA published on a journal from the American Chemical Association which, in our opinion, provides enough support for our statement. We kindly ask the reviewer to clarify why they think this reference is not valid and perhaps suggest an alternative.

L76: it is still Thaumarchaeota.

We agree with the reviewer in this point. All mentions of “Class Nitrososphaeria” and “former phylum Thaumarchaeota” have been changed to “phylum Thaumarchaeota”.

L81: it is needed information on how the genome was analyzed and how to prepare, collect, analyze the genome sample.

We thank the reviewer for pointing this out. We have now included a thorough description of how the four novel MAGs were obtained:

Sampling and metagenome sequencing

Ten soil samples were obtained in July 2017 across an area of alpine tundra in Rásttigáisá, Norway (69°59’N, 26°15’E, 700 m.a.s.l.). DNA was extracted from the mineral layer (10–15 cm depth) with the PowerSoil DNA Isolation kit (QIAGEN, Venlo, Netherlands) according to the manufacturer’s instructions. Paired-end metagenomic sequencing was done using the Illumina NextSeq500 platform (Illumina, San Diego, CA, USA) at the DNA Sequencing and Genomics Laboratory (Institute of Biotechnology, University of Helsinki).

Metagenome assembling and binning

Removal of adapter sequences and low-quality base calls (Phred score < 28) was done with Cutadapt v1.10 (Martin, 2011) and sequences were assembled with MEGAHIT v1.1.1 setting a minimum contig length of 1,000 bp (Li et al., 2015). Samples were assembled individually and as one co-assembly of all pooled samples. Manual MAG binning was done with anvi’o v6.0 (Eren et al., 2015) according to Pessi et al. (2022, pre-print). In brief, Prodigal v2.6.3 (Hyatt et al., 2010) was used to predict gene calls and single-copy genes were identified with HMMER v.3.2.1 (Eddy, 2011). Bowtie v2.3.5 (Langmead and Salzberg, 2012) and SAMtools v1.9 (Li et al., 2009) were used to map the quality-filtered Illumina reads to the contigs. Contigs were then manually binned into MAGs based on differential coverage and tetranucleotide frequency using the anvi-interactive interface of anvi’o v6.0. MAGs were manually inspected and refined using the anvi-refine interface of anvi’o v6.0.

Genomic dataset Metagenome-assembled genomes assigned to the UBA14052 lineage

MAGs were classified based on 122 archaeal and 120 bacterial single-copy genes with GTDB-Tk v1.3.0 (Chaumeil et al., 2019) and the GTDB release 05-RS95 (Parks et al., 2018, 2020). MAGs assigned to the genus “UBA10452” in the order Nitrososphaerales (“terrestrial group I.1b”), phylum Thaumarchaeota (Rinke et al., 2021), were selected for downstream analyses (Table 1, Suppl. Table S1). In addition, we analysed other eight UBA10452 MAGs available on GenBank and GTDB release 95 (Parks et al., 2018, 2020). These included six MAGs from permafrost soil in Canada (Chauhan et al., 2014; Parks et al., 2017), one MAG from polar desert soil in Antarctica (Ji et al., 2017), and one MAG from tundra soil in Finland (Pessi et al., 2022, pre-print).

Methods, lines 77–104

We invite the reviewer to look at the remaining of the Methods section for information on how the genomes were analysed. Finally, we would like to point out that all the code used to run these analyses has now been added to GitHub:

Genomic assemblies can be found in GenBank/ENA under the accession numbers listed in Table 1. MAGs generated in this study have been submitted to ENA (BioProject PRJEB49283). All the code used can be found in https://github.com/ArcticMicrobialEcology/candidatus-nitrosopolaris.

Data availability, lines 464–467

Figure 2: there are not many isolated AOA genomes; why was it not included? And are those LB2_1, LB3_1, and so on, belong to the genus Nitrosocosmicus?

We have included in our phylogenomic analysis all genomes from the family Nitrososphaeraceae that are available on GenBank, which includes both isolate and environmental genomes. Apart from Nitrosopumilus maritimus SCM1 which was used as outgroup, we have not included other non-Nitrososphaeraceae AOA in our analysis as i) we believe that they are not relevant for assessing the phylogenetic placement of our taxon and ii) a complete revision of the phylogeny of AOA is beyond the scope of our study.

We thank the reviewer for bringing our attention to the “LB/YB/MB” genomes. Indeed, they are not part of Nitrosocosmicus but instead represent the newly proposed Candidatus Nitrosodeserticola (Hwang et al. 2021). We have thus modified the text to correct the taxonomic affiliation of these genomes, e.g.:

Phylogenomic analysis based on 59 single-copy genes placed the UBA10452 MAGs as a distinct lineage outside Nitrososphaera, Ca. Nitrosocosmicus, and Ca. Nitrosodeserticola, the three described genera in the family Nitrososphaeraceae (Stieglmeier et al., 2014; Lehtovirta-Morley et al., 2016; Hwang et al., 2021) (Fig. 2a; Suppl. Fig. S2).

Results, lines 216–219

Figure 2 and Suppl. Figure S3 have also been modified.

L212-213 in Figure 3: It would not be matched because of the low 16s rRNA gene ratio in the metagenome.

We confess we have struggled with this comment, as it is not clear to us what would not be matched to what. We would like to clarify that this figure is based on metagenomic read recruitment against full genomic sequences, so it is not at all related to the 16S rRNA gene.

L222: 8.6% ratio is so high. Please recheck it.

We thank the reviewer for this observation. We have redone the read recruitment analysis and have confirmed that the values are correct. We would like to point out that the permafrost samples in particular represent very simple communities in extremely oligotrophic environments, so these high values do not seem so surprising in our opinion. We have, however, modify the text and figures to emphasize that we are dealing with the proportion of reads in the metagenomic datasets recruited by the MAGs rather than actual abundances in the environment:

UBA10452 MAGs were most abundant in the permafrost from nutrient-poor (C: 1.0%, N: 0.1%) mineral cryosoils in Nunavut, Canadian Arctic, where they recruited up to 8.6% of the reads in each sample (Fig. 1c).

Results, lines 198–200

L230, 234-235: It seems like to unusually high ratio. Is it this like high? If so, why the reason?

This is the same issue as above and has also been confirmed by redoing the analyses. We would like to emphasise that, in this case, we are dealing with amplicon sequencing libraries using primers specific to archaea. Thus, these values do not represent the total community but rather the archaeal fraction of the communities. Again, given that these are simple communities in oligotrophic environments, we do not find so surprising to have the archaeal community essentially dominated by one taxon. We thank the reviewer for bringing this to our attention and have modified the text and figures to clarify this issue:

The proportion of reads matching the UBA10452 lineage was above 50% of the archaeal 16S rRNA gene sequences in 70 of these sites and reached values as high as 88.8% (Suppl. Fig. S2b).

Results, lines 212–214

L244: I don’t know why all separated into two contigs? There is no detection in other contigs?

This is a very interesting observation. It is indeed surprising that all 12 MAGs show the same pattern of amo genes separated across two contigs. This is likely due to an assembly issue, probably related to some conserved gene or repetitive region at the edges of the contigs.

L250-253: it is not clear whether it is about ammonia or ammonium?

We thank the reviewer for pointing this out. The sentence has now been corrected:

In addition to amo genes, Ca. Nitrosopolaris also encodes an ammonium transporter of the Amt family involved in the uptake of extracellular ammonium .

Results, lines 276–278

L309-312: There is the threshold for defining genus level (for ANI and AAI, also for 16S rRNA gene), as far as I know correctly. Please recheck it.

We thank the reviewer for the suggestion. We have now used a 65% AAI threshold for genus-level delineation as proposed by Konstantinidis et al. 2017:

Separation of UBA10452 is also supported by AAI and 16S rRNA gene analyses. UBA10452 MAGs share 59.1% ± 1.9, 53.0% ± 1.1, and 53.8% ± 0.9 AAI with Nitrososphaera, Ca. Nitrosocosmicus, and Ca. Nitrosodeserticola, respectively (Fig. 2b), all of which are below the 65% AAI threshold commonly used to delineate microbial genera (Konstantinidis et al., 2017).

Results, lines 219–223

In regards to the 16S rRNA gene, this has now been added:

At the 16S rRNA gene level, UBA10452 MAGs are 94.8% ± 1.2 and 95.4% ± 0.2 similar to Nitrososphaera and Ca. Nitrosocosmicus, respectively (Suppl. Fig. S1)(Suppl. Fig. S3). These values are in the limit of the 95% threshold for genus delineation proposed by Rosselló-Móra and Amann (2015), but are well below the median 16S rRNA gene similarity observed between related genera across different microbial phyla (96.4%; Yarza et al., 2014). Comparison with Ca. Nitrosodeserticola was not possible due to the lack of a 16S rRNA gene sequence from this genus.

Results, lines 223–229

The original sentence highlighted by the reviewer has, however, been removed to make the discussion more streamlined.

Table S1. It is not possible to see the table at a glance.

The table was provided as a separate xls (MS Excel) file, but it seems it might have been converted to a different format by the journal after submission. We invite the reviewer to ask the editor for the original xls file which should be made available in this format upon publication.

**Reviews of manuscript FEMSMC-2021-061.r1 (Second Draft)**

**Reviewer 1 - Anne Daebeler (Biology Centre CAS, Soil and Water Research Infrastructure)**

**Recommendation: Minor revision**

I want to thank the authors for carefully revising the manuscript. A few minor comments are listed below.

I was specifically asked by the handling editor to comment on the suggestions and remarks made by reviewer 2. Therefore, I have read the responses the authors gave to reviewer 2 carefully and also inspected their changes to the manuscript based on the suggestions made by reviewer 2. I agree with all changes made and believe the authors have answered adequately to the raised concerns. Specifically, in my view the description of a new Candidatus genus complies with common practices in the field and is done with the necessary caution here. Certainly a culture is needed to name the genus systematically, but this is not for this journal and goes much beyond the intention of this work. Giving a Candidatus name is justified and should not be revoked from the manuscript.

- line 78: Spelling mistake -> thermophilic

- line 82-83, 115 and elsewhere: The authors need to decide whether they comply with the GTDB or the NCBI taxonomy. Both is fine by me, but be consistent and cite accordingly. Here, if Thaumarchaeota are meant to be used as the phylum name, the reference to Rinke is wrong.

- Fig. 2a: It is a shame that the bootstrap values have disappeared. Please provide a tree with bootstrap values (or indications thereof like with white/grey/black circles on nodes for >70/ >80/ >90 values) so that the readers can have confidence in the phylogenetic analysis. Since the tree in the supplement has this information, it should be easy to fix this issue.

- Fig. 3b: Same as above. Please provide bootstrap values for the tree or present a consensus tree of 2-3 different treeing methods. Otherwise it is difficult to assess the confidence level for the phylogenetic analysis.

- line 579: I assume the authors meant to say '.. habours the complete set of AMO genes,...' The mere presence of a full amoA gene is not enough. In this context I would to draw the authors attention to the following pre-print: https://www.biorxiv.org/content/10.1101/2022.04.06.487334v1.full. Here the authors present data that AMO may consist of more subunits in AOA than previously recognized. This work has not undergone peer-review and should be discussed carefully, but is very intriguing. Probably, it is worthwhile to check the presence and genomic arrangement of the newly proposed AMO subunits Y and Z in the MAGs of this study.

**Author's Response to Decision Letter for (FEMSMC-2021-061.r1)**

Dear Editor Dr. Jana Jass,

We thank you and reviewer 1 for the comments on our manuscript “Candidatus Nitrosopolaris, a genus of putative ammonia-oxidizing archaea with a polar/alpine distribution”.

We have carefully revised the sections Reviewer 1 had comments on. Specifically, we have revised the phylogenetic trees with bootstrap values to have confidence in the phylogenetic analysis. In addition, we have checked our MAGs for the presence of the proposed amoXY genes as suggested by the reviewer and have added discussion on this to the revised manuscript. Last, we have carefully revised the sections on taxonomy.

We hope you will find our manuscript suitable for publication on FEMS Microbes.

On behalf of the co-authors,

Jenni Hultman, PhD

Docent (Adjunct Professor) in Microbiology and Academy Research Fellow

Department of Microbiology, University of Helsinki, Finland

1 Reviewer: 1

I want to thank the authors for carefully revising the manuscript. A few minor comments are listed below.

I was specifically asked by the handling editor to comment on the suggestions and remarks made by reviewer 2. Therefore, I have read the responses the authors gave to reviewer 2 carefully and also inspected their changes to the manuscript based on the suggestions made by reviewer 2. I agree with all changes made and believe the authors have answered adequately to the raised concerns. Specifically, in my view the description of a new Candidatus genus complies with common practices in the field and is done with the necessary caution here. Certainly a culture is needed to name the genus systematically, but this is not for this journal and goes much beyond the intention of this work. Giving a Candidatus name is justified and should not be revoked from the manuscript.

We thank the reviewer once again for their interest in our work and for advising with the issues raised by the other reviewer. Please find below our point-by-point response.

Additionally, we are happy to add to the previous comment about adding “pre-print” to our other manuscript: the manuscript has gone through peer-review and has now been accepted in Environmental Microbiome, so the reference has been changed from “pre-print” to “published”.

- line 78: Spelling mistake -> thermophilic

Thank you for pointing this out, the typo has been fixed.

- line 82-83, 115 and elsewhere: The authors need to decide whether they comply with the GTDB or the NCBI taxonomy. Both is fine by me, but be consistent and cite accordingly. Here, if Thaumarchaeota are meant to be used as the phylum name, the reference to Rinke is wrong.

Originally we have used the GTDB taxonomy, i.e. phylum Thermoproteota as proposed by Rinke et al. However, we agreed with Reviewer 2 at the previous round of review that Thaumarchaeota is still the officially recognized name and have thus changed to phylum Thaumarchaeota throughout the manuscript. In fact, most of the citations to Rinke et al. in the text are actually refering to the specific UBA10452 lineage (described in Rinke et al.), not to the whole phylum. We do however agree that in some parts this can be misleading, and have thus revised several parts:

Moreover, the growing use of genome-resolved metagenomics has resulted in the identification of tens of novel, currently uncultured lineages in the phylum Thaumarchaeota.These lineages are phylogenetically distinct from both formally described and Candidatus taxa and are normally labelled with placeholder alphanumeric identifiers (e.g., the Nitrososphaerales genus “UBA10452”; Rinke et al., 2021). [Introduction, lines 57–61]

The remaining amoA gene was binned into a metagenome-assembled genome (MAG) assigned to the genus “UBA10452”, an uncharacterized archaeal lineage in the order Nitrososphaerales (Rinke et al., 2021). [Introduction, lines 70–72]

MAGs assigned to the genus “UBA10452” in the order Nitrososphaerales (Rinke et al., 2021), were selected for downstream analyses (Table 1, Suppl. Table S1). [Methods, lines 99–100]

We applied a genome-resolved metagenomics approach to data obtained from tundra soils in Rásttigáisá, Norway, and recovered four MAGs assigned to the genus “UBA10452”, an uncultured lineage in the order Nitrososphaerales (“terrestrial group I.1b”), phylum Thaumarchaeota. [Results, lines 188–199]

In a recent metagenomic investigation of tundra soils in northern Finland (Pessi et al., 2022, pre-print), we have manually binned and curated a MAG belonging to the genus “UBA10452”, an uncultured and largely uncharacterized lineage in the order Nitrososphaerales (“terrestrial group I.1b”) of the phylum Thaumarchaeota. [Discussion, lines 363–366]

- Fig. 2a: It is a shame that the bootstrap values have disappeared. Please provide a tree with bootstrap values (or indications thereof like with white/grey/black circles on nodes for >70/ >80/ >90 values) so that the readers can have confidence in the phylogenetic analysis. Since the tree in the supplement has this information, it should be easy to fix this issue.

We thank the reviewer for bringing our attention to this issue. Given that the bootstraped tree was made available in Suppl. Fig. S2, we have decided to have Fig. 2 as a more simplified representation. However we agree that bootstrap values are important and have brought them back to Fig. 2, and Suppl. Fig. S2 was also improved.

- Fig. 3b: Same as above. Please provide bootstrap values for the tree or present a consensus tree of 2-3 different treeing methods. Otherwise it is difficult to assess the confidence level for the phylogenetic analysis.

As requested, bootstrap values have been added to Fig. 3 as well:

- line 579: I assume the authors meant to say '.. habours the complete set of AMO genes,...' The mere presence of a full amoA gene is not enough. In this context I would to draw the authors attention to the following pre-print: https://www.biorxiv.org/content/10.1101/2022.04.06.487334v1.full. Here the authors present data that AMO may consist of more subunits in AOA than previously recognized. This work has not undergone peer-review and should be discussed carefully, but is very intriguing. Probably, it is worthwhile to check the presence and genomic arrangement of the newly proposed AMO subunits Y and Z in the MAGs of this study.

We thank the reviewer for bringing our attention to this. Indeed, we meant to say the complete set of amo genes. This has now been corrected:

Ca. Nitrosopolaris harbours the complete set of amoABCX genes responsible for chemolithotrophic growth via ammonia oxidation (Lehtovirta-Morley, 2018). [Discussion, lines 392–393]

As suggested, we have also checked the MAGs for the presence of the proposed amoXY genes and have added this to the manuscript:

We used BLASTP v2.10.1 to compare the amino acid sequences of genes identified as amoA, amoB, amoC, or amoX to the RefSeq (O’Leary et al., 2016) and Swiss-Prot (The UniProt Consortium, 2019) databases, and BLASTN v2.10.1 (Camacho et al., 2009) to compare amoA genes against the nt database and the curated amoA database of Alves et al. (2018). BLASTP v2.10.1 was also used to identify the putative amoYZ genes proposed recently by Hodgskiss et al. (2022, preprint). [Methods, lines 135–140]

Annotation of protein-coding genes revealed that Ca. Nitrosopolaris harbours the amoA, amoB, amoC, and amoX genes encoding the enzyme ammonia monooxygenase (Amo) which catalyses the oxidation of ammonia to hydroxylamine (Fig. 3a, Suppl. Fig. S4, Suppl. Table S2). Putative homologues of the amoYZ genes proposed by Hodgskiss et al. (2022, preprint) were found in all Ca. Nitrosopolaris MAGs, although with relatively low amino acid similarity for amoZ (mean ± sd: amoY, 82.6% ± 16.6%; amoZ, 32.3% ± 1.38%). [Results, lines 268–273]

With few exceptions, putative amoYZ genes (Hodgskiss et al., 2022, preprint) were located adjacent to each other but in a different contig from the other amo genes. In some MAGs, this contig contained the ammonium transporter amt gene at 9–10 genes away from the amoYZ cluster. [Results, lines 292–295]

Ca. Nitrosopolaris harbours the complete set of amoABCX genes responsible for chemolithotrophic growth via ammonia oxidation (Lehtovirta-Morley, 2018). In addition, putative homologues of the recently proposed amoYZ genes were found, although with low confidence for amoZ. These genes have been linked to two potential additional Amo subunits in Nitrososphaera viennensis and other AOA, but further studies are needed to confirm this association (Hodgskiss et al., 2022, preprint). [Discussion, lines 392–397]
